# Heat Stress Triggers Nuclear Invagination and Spatial Compartmentalization of Protein Metabolism

**DOI:** 10.1111/cpr.70196

**Published:** 2026-03-12

**Authors:** Zhi‐Hao Zhang, Yuan‐Tao Qiu, Jing‐Yue Cao, Yi‐Feng Huang, Ye‐Lin Lan, Yu‐Li Zhao, Fu Wu, Chen‐Long Wang, Guo‐Run Tang, Jing‐Yun Ji, Zhang Zhang, Guan‐Zheng Luo

**Affiliations:** ^1^ MOE Key Laboratory of Gene Function and Regulation, Guangdong Province Key Laboratory of Pharmaceutical Functional Genes, State Key Laboratory of Biocontrol, School of Life Sciences Sun Yat‐Sen University Guangzhou China; ^2^ Innovation Center for Evolutionary Synthetic Biology Sun Yat‐Sen University Guangzhou China; ^3^ Institute of Advanced Studies Hong Kong Sun Yat‐Sen University Hong Kong SAR China

## Abstract

Heat stress is a common challenge for cells, causing multiple types of cellular damage while triggering complex stress responses, including the highly conserved mechanism known as the heat shock response (HSR). However, the subcellular coordination of these stress responses remains poorly understood. In this study, we identify reversible nuclear morphological changes under heat stress, characterised by kidney‐shaped invaginations. These nuclear invaginations are associated with intermediate filament collapse and the regional clustering of organelles. Through immunofluorescence imaging and proteomic analysis, we further reveal that nuclear invagination regions function as specialised compartments where newly synthesised proteins are concentrated and protein degradation demand is heightened. Moreover, this compartmentalization is not only essential for cellular adaptation and recovery from heat stress but also correlates with the differential heat tolerance across cell lines. Our findings highlight a previously unappreciated mechanism by which cells spatially reorganise protein metabolism to optimise stress responses, providing new insights into cellular stress adaptation.

## Graphical abstract

Cells adapt heat stress to shape a nuclear invagination region function as “protein metabolism hotspots”, where both protein production and degradation are enhanced.

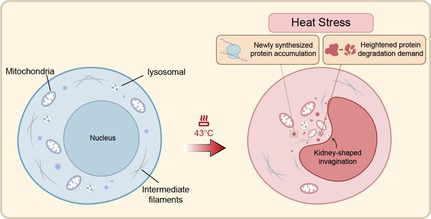

## 1 Introduction

As one of the most common environmental stressors encountered by organisms, heat stress has profound impacts on cellular metabolism and physiological activities. Cellular responses to heat stress depend on multiple factors, such as stress intensity, cell type and microenvironment [1, 2]. Elevated temperature triggers a cascade of cellular responses, including cytoskeleton rearrangements [3, 4, 5, 6], protein aggregation [7, 8, 9, 10, 11], organelle mislocalization [12, 13] and alterations in membrane properties [14, 15, 16]. These changes collectively challenge cellular homeostasis and necessitate robust stress response mechanisms. Concurrently, molecular signalling pathways, particularly the heat shock response (HSR), are activated to maintain cellular homeostasis [17]. At supraphysiological temperatures, hyperthermia effectively kills cells and has been applied as an adjuvant therapy to enhance cancer treatment efficacy [18, 19, 20, 21]. Consequently, research on heat stress offers valuable insights for developing novel cellular protection strategies and therapeutic approaches, with broad implications for biomedical research and clinical applications.

Research on heat stress has been marked by the landmark discovery of heat shock genes [22, 23, 24]. Under heat stress conditions, heat shock transcription factor 1 (HSF1) undergoes oligomerization and nuclear translocation, driving transcriptional activation of heat shock proteins (HSPs) [25, 26, 27]. Acting as molecular chaperones, HSPs maintain protein homeostasis through diverse mechanisms, including assisting protein folding, preventing protein aggregation and promoting the degradation of misfolded proteins [28, 29]. Although the heat shock response is a cornerstone of cellular defence, it is now clear that cells deploy a multifaceted arsenal of stress responses to combat heat stress. For example, long non‐coding RNAs such as Alu are upregulated during heat stress and act as trans‐acting transcriptional repressors. By interacting with RNA polymerase II (Pol2) and occupying promoters in transcriptional complexes, they lead to widespread transcriptional suppression [30, 31, 32]. At the translational level, global inhibition is mediated through eIF2 phosphorylation by its kinases during heat stress, resulting in blocked translation initiation [33, 34, 35, 36]. Heat stress also induces various subcellular changes, including the assembly of stress granules that sequester numerous mRNAs and translation initiation components, further impeding translation [37, 38, 39, 40]. Additionally, heat stress activates the ubiquitin‐proteasome system, accelerating the degradation of misfolded proteins, clearing abnormal proteins and stress granules and regulating cellular autophagy [41, 42, 43, 44, 45, 46].

Despite significant advances in understanding molecular pathways responding to heat stress, the spatial organisation and coordination of these responses within the cell remains largely unexplored. In this study, we report the nuclear invagination phenotype induced by heat stress and systematically characterise its graduality, generality and reversibility. Our analysis revealed that this phenotype is accompanied by subcellular responses, including the collapse of the intermediate filament network and the clustering of mitochondria and lysosomes. Using immunofluorescence imaging and proteomic analysis, we investigated the function of nuclear invagination regions and found that these reversible invaginations, accompanied by spatial reorganisation of organelles, serve as specialised compartments that concentrate newly synthesised proteins and exhibit heightened demands for protein degradation during heat stress. Furthermore, we explored the potential biological significance of nuclear invagination. Our results show that inhibiting the formation of nuclear invagination impairs cell proliferation and survival under heat stress and the extent of nuclear invagination formation across cell lines correlates with their heat tolerance. We propose that nuclear invagination represents a novel subcellular‐level heat stress response that facilitates cellular adaptation.

## 2 Results

### 2.1 Nuclear Invagination Phenotype Induced by Heat Stress

**FIGURE 1:**
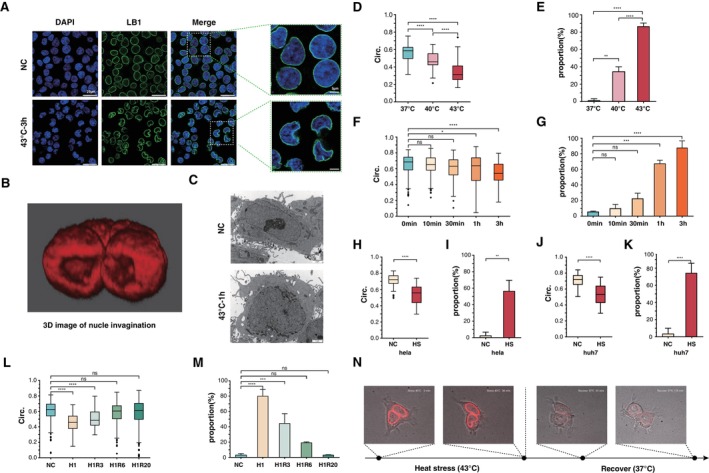
Heat stress induces dynamic and reversible nuclear invagination phenotype. (A) Immunofluorescence of HEK293T cells stained with DAPI (nuclei, blue) and Lamin B1 (nuclear membrane, green), showing nuclear morphology alterations before (Untreated Cells, NC) and after heat treatment (43°C–3 h). White boxes indicate regions magnified in right panels. Scale bars: 25 μm (left), 5 μm (right). (B) Three‐dimensional reconstruction of cells exhibiting nuclear invaginations generated from Lamin B1 channel signals. (C) Transmission electron microscopy reveals cellular ultrastructural alterations in cells under basal (NC, up) and heat‐stressed (43°C, 1 h; down) conditions. Scale bar: 2 μm. (D, E) Quantification of nuclear morphology under different heat stress temperature gradients (37°C, 40°C, 43°C; 2 h treatment). (D) Nuclear circularity. (E) Invagination frequency. (*n*_37°C_ = 72, *n*_40°C_ = 59, *n*_43°C_ = 78). (F, G) Quantification of nuclear morphology under different heat stress duration gradients (43°C; 0 min, 10 min, 30 min, 1 h, 3 h treatment). (F) Nuclear circularity. (G) Invagination frequency. (*n*_0min_ = 127, *n*_10min_ = 144, *n*_30min_ = 153, *n*_1h_ = 138, *n*_3h_ = 111). (H, I) Quantification of nuclear morphology in HeLa cells under heat stress. (43°C, 3 h). (H) Nuclear circularity. (I) Invagination frequency. (*n*_NC_ = 40, *n*_HS_ = 60). (J, K) Quantification of nuclear morphology in Huh7 cells under heat stress. (43°C, 3 h). (J) Nuclear circularity. (K) Invagination frequency. (*n*_NC_ = 25, *n*_HS_ = 35). (L, M) Recovery kinetics of nuclear morphology after 1 h heat stress (43°C). (Timepoints H1, H1R3, H1R6, H1R20 represent 1 h heat stress, followed by 3 h, 6 h and 20 h recovery at 37°C, respectively.) (H) Nuclear circularity. (I) Invagination frequency. (*n*_NC_ = 208, *n*_H1_ = 91, *n*_H1R3_ = 80, *n*_H1R6_ = 206, *n*_H1R20_ = 302). (N) Representative time‐lapse frames from live‐cell imaging (Video S1) capturing the dynamic process of nuclear invagination formation (during 43°C stress, frame at 36 min) and subsequent recovery (during 37°C recovery, frames at 35 min and 135 min). Statistical analysis: Data in D‐M were analysed using one‐way ANOVA followed by Tukey's post hoc test for multiple comparisons. *p* values were represented by standard labels: Ns. not significant, *p < 0.05, **p < 0.01, ***p < 0.001, ****p < 0.0001.

To investigate subcellular responses to heat stress, we subjected HEK293T cells to 43°C heat treatment for 3 h. Surprisingly, immunofluorescence staining for the nuclear envelope marker Lamin B1 (LB1) revealed a novel nuclear phenotype characterised by kidney‐shaped invaginations in a considerable proportion of cells (Figure 1A). Although nuclear morphology is known to be altered in certain pathological contexts [47, 48, 49, 50], heat stress‐induced nuclear morphological changes have not been previously reported and remain largely uncharacterized. To better characterise the nuclear invagination phenotype, we utilised three‐dimensional imaging to visualise nuclei displaying this phenotype (Figure 1B). Furthermore, transmission electron microscopy revealed ultrastructure features of cells with nuclear invaginations, including nuclear shrinkage and invagination (Figure 1C).

To facilitate quantitative and large‐scale analysis of nuclear invagination phenotypes, we employed circularity (Circ = 4π × S/C^2^) as a quantitative metric that lower circularity values indicate increased nuclear irregularity and invagination. To determine the optimal conditions for inducing nuclear invaginations, we assessed the effects of varying temperature and duration of heat stress. Increasing the temperature from 40°C to 43°C resulted in a progressive decrease in nuclear circularity and a corresponding increase in the proportion of cells exhibiting nuclear invaginations (Figure 1D,E; Figure S1A). Similarly, prolonging the duration of 43°C heat stress led to a time‐dependent decrease in nuclear circularity, with a significant reduction observed after 1 h, at which point approximately two‐thirds of cells displayed the invagination phenotype (Figure 1F,G; Figure S1B). Collectively, these results demonstrate that nuclear invagination phenotypes are both temperature‐ and time‐dependent, with HEK293T cells developing pronounced nuclear invagination phenotypes after 1 h of stimulation at 43°C. Given that cellular stress responses can be highly context‐dependent, we investigated whether nuclear invagination represents a cell type‐specific phenomenon or a general cellular adaptation. To address this, we examined two additional human cell lines with distinct tissue origins. Under 43°C heat treatment for 3 h, both HeLa and Huh7 cells exhibited nuclear invaginations, characterised by significantly reduced nuclear circularity and elevated proportions of cells with nuclear invagination (Figure 1H–K; Figure S1C,D).

To exclude the possibility that the nuclear invagination was an apoptosis‐related phenotype [1, 2, 18, 20], we examined whether this phenotype is reversible. Flow cytometry analysis revealed that treatment at 43°C for up to 3 h did not induce significant apoptosis (Figure S1E). We then allowed the heat‐treated cells to recover and quantified the proportion of cells with nuclear invagination and changes in nuclear circularity at different time points (Figure S1F). Under the condition of 43°C for 1 h, the proportion of cells exhibiting nuclear invagination gradually decreased with increasing recovery time, accompanied by progressive recovery of nuclear circularity. After 6 h of recovery, nuclear circularity showed no significant difference compared to control cells, and the invagination phenotype completely disappeared after 20 h of recovery (Figure 1L,M).

To control sample heterogeneity and cell proliferation artefacts during recovery, we established an mCherry‐LMNB1 cell line to directly visualise the nuclear envelope in live cells (Figure S1G). Live‐cell imaging confirmed the heat‐induced nuclear invagination phenotype, ruling out immunofluorescence artefacts (Figure S1H). We then performed live‐cell time‐lapse imaging and captured the process of nuclear invagination formation under heat stress and its subsequent disappearance during recovery (Figure 1N; Video S1). These results confirmed cell viability during invagination and the reversible nature of the phenotype, implying a functional role for this reversible nuclear morphological change in the cellular heat stress response.

### 2.2 Nuclear Invagination Is Accompanied by Cytoskeletal Collapse and Organelle Clustering

**FIGURE 2:**
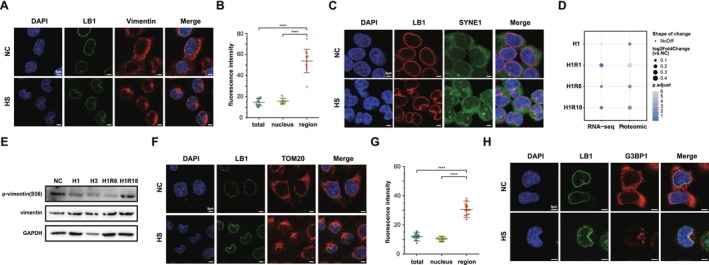
Nuclear invagination is accompanied by intermediate filament remodelling and organelle redistribution. (A) Immunofluorescence of Hela cells stained with anti‐Vimentin (intermediate filament, red), Lamin B1 (nuclear membrane, green) and DAPI (nuclei, blue), showing intermediate filament reorganisation following heat stress (HS, 43°C for 3 h) compared to untreated cells (NC). Scale bars: 5 μm. (B) Quantification of Vimentin fluorescence intensity, showing enrichment in nuclear invagination regions. (*n* = 12). (C) Immunofluorescence of HEK293T cells stained with anti‐SYNE1 (LINC complex, green), Lamin B1 (nuclear membrane, red) and DAPI (nuclei, blue), showing SYNE1 aggregation at nuclear invaginations following heat stress (HS, 43°C for 1 h) compared to untreated cells (NC). Scale bars: 5 μm. (D) Temporal profiling of Vimentin expression dynamics during heat stress and recovery, assessed by RNA‐sequencing (left) and proteomics analysis (right). The dot plot shows the log_2_(Fold Change) of Vimentin expression relative to the NC condition (Conditions H1, H1R1, H1R6, H1R18 represent 1 h heat stress at 43°C, followed by 1 h, 6 h and 18 h recovery at 37°C, respectively). Both analyses indicate no significant change in Vimentin expression levels across these conditions. (E) Western blot analysis showing the phosphorylation status of Vimentin at Ser‐56. Lysates were collected from NC, 1 h HS (H1), 3 h HS (H3) and recovery time points (1 h HS followed by 6 h recovery, H1R6; 1 h HS followed by 18 h recovery, H1R18). (F) Immunofluorescence of Hela cells stained with anti‐TOM20 (mitochondria, red), Lamin B1 (nuclear membrane, green) and DAPI (nuclei, blue), showing mitochondrial redistribution following heat stress (HS, 43°C for 3 h) compared to untreated cells (NC). Scale bars: 5 μm. (G) Quantification of TOM20 fluorescence intensity, showing mitochondrial enrichment in nuclear invagination regions. (*n* = 17). (H) Immunofluorescence of Hela cells stained with anti‐G3BP1 (stress granule, red), Lamin B1 (nuclear membrane, green) and DAPI (nuclei, blue), showing the formation and accumulation of stress granules following heat stress (HS, 43°C for 3 h) compared to untreated cells (NC). Scale bars: 5 μm. Statistical analysis: Data in (B, G) were analysed using one‐way ANOVA followed by Tukey's post hoc test for multiple comparisons. The significance labels are defined in the Figure 1 legend.

Given the established impact of heat stress on cellular physiology [17, 51], we asked whether the nuclear invagination phenotype was accompanied by other subcellular alterations. The cytoskeleton, composed of microfilaments, microtubules and intermediate filaments, is critical for maintaining cell shape, mechanical integrity and organelle positioning [52, 53]. Previous studies have shown that heat stress can alter and even disrupt the cytoskeleton [3, 6, 12]. To facilitate visualisation of cytoplasmic changes, we examined the three cytoskeletal components during nuclear invagination in HeLa cells, which have a lower nuclear‐to‐cytoplasmic ratio (Figure 2A; Figure S2A,C). Interestingly, while heat stress induced cell and nuclear morphological changes, the distribution of microfilaments and microtubules remained largely unaffected (Figure S2A–D). In contrast, intermediate filaments exhibited a dramatic reorganisation upon heat stress that, whereas normally distributed throughout the cytoplasm, the intermediate filament network collapsed and clustered around the nucleus (Figure 2A,B).

Given the intimate physical connection between the cytoskeleton and the nuclear envelope [54, 55], we hypothesised that this pronounced perinuclear condensation of intermediate filaments could exert localised mechanical forces on the nucleus, potentially contributing to invagination. To explore this possibility, we investigated the linker of nucleoskeleton and cytoskeleton (LINC) complex and the nuclear pore complex (NPC) by immunofluorescence. While the inner nuclear membrane protein SUN1 and the nucleoporin NUP153 did not exhibit marked redistribution (Figure S2E,F), we observed that SYNE1 (Nesprin‐1), the cytoplasmic‐facing component of the LINC complex, formed distinct puncta adjacent to invagination sites in a subset of cells (Figure 2C). This specific localization pattern suggests a structural and potentially mechanical linkage between the cytoskeleton, the LINC complex and the deforming nuclear envelope. Whether this linkage represents a driving force or a concomitant structural adaptation, however, remains to be determined.

To investigate the mechanism underlying the observed intermediate filament network collapse, we performed RNA‐seq and proteome analysis before and after heat stress. Surprisingly, no significant changes were observed in intermediate filament expression at either the transcriptional or translational levels during heat stress or recovery (Figure 2D), suggesting that the collapse of the intermediate filament network was not driven by expression changes. We hypothesised that the alterations in the intermediate filament network might instead be influenced by changes in their solubility and assembly properties. Given that intermediate filament solubility is regulated by phosphorylation [56, 57], we first identified the phosphorylation sites on vimentin through proteomics analysis. We then validated the most abundant site, S56, by Western blot and found that S56 phosphorylation decreased after heat stress and returned to baseline levels after 20 h of recovery (Figure 2E). These results suggest that the changes in the intermediate filament network may be regulated by their phosphorylation levels during heat stress.

The intermediate filament network is known to be associated with organelle positioning [58, 59, 60, 61, 62]. Therefore, we investigated whether nuclear invagination is accompanied by changes in organelle localization. During heat stress, as the intermediate filament network collapsed, we observed mitochondria accumulated in the nuclear invagination regions (Figure 2F,G). Following recovery, the mitochondrial accumulation was reversed, along with the disappearance of nuclear invagination (Figure S2G), suggesting potentially altered metabolic activity in these regions. Through G3BP1 staining, we also observed the formation and accumulation of stress granules during heat stress (Figure 2H). Additionally, nucleoli underwent disassembly and became more diffuse concurrent with nuclear invagination (Figure S2H). These coordinated changes in organelles and subcellular structures underscore the complexity of the cellular response to heat stress and suggest a potential functional link between nuclear invaginations, intermediate filament remodelling and organelle redistribution.

### 2.3 Assessing the Candidate Regulators in Heat Stress‐Induced Nuclear Invagination

The coordinated reorganisation of multiple subcellular structures prompted us to investigate potential upstream molecular requirements for nuclear invagination. We conducted a systematic, hypothesis‐driven assessment to evaluate the requirement of candidate genes spanning distinct functional categories implicated in nuclear integrity, cytoskeletal dynamics and stress signalling. Using CRISPR‐Cas9 or overexpression constructs, we generated cell lines with perturbations in a defined set of candidate genes. These included components of the LINC complex (SUN1, SYNE1, SYNE2, SYNE3, SYNE4), chromatin remodelers (SMARCA4), the nuclear pore complex (NUP153, RANBP2), the cytoskeleton (ACTB, TUBB, VIM), nucleolar proteins (EIF6, FBL), metabolism factors (EIF2A, EIF4A, PSMD1), as well as overexpression of molecular chaperones (DNAJB1), the cytoskeletal component (VIM) and nuclear lamins (LMNA, LMNB1). The efficiency of knockdown or overexpression was validated by RT‐qPCR for each target, though the knockdown efficiency varied and was suboptimal for some essential genes (Figure S3A,B). Notably, within the limits of this screening approach, none of these individual perturbations resulted in a significant or reproducible alteration of the nuclear invagination phenotype (Figure S3C,D). It is noteworthy that several components which exhibit dramatic spatial reorganisation during invagination—such as vimentin, SYNE1 and nucleolar markers—were themselves dispensable for the initiation of the phenotype when individually perturbed.

We also employed specific pharmacological inhibitors to assess the potential involvement. Pretreatment with inhibitors targeting the JNK (SP600125), p38 (SB203580), or ERK (U0126) MAP kinase pathways also failed to prevent nuclear invagination formation upon heat shock (Figure S3C,D). These results collectively suggest that the redistribution of subcellular structures may be a concurrent or downstream consequence of a more global cellular restructuring process, rather than its upstream cause. Alternatively, it may reflect a high degree of functional redundancy within these structural networks.

### 2.4 Nuclear Invagination Accumulate Newly Synthesised Proteins for Localised Metabolism

**FIGURE 3:**
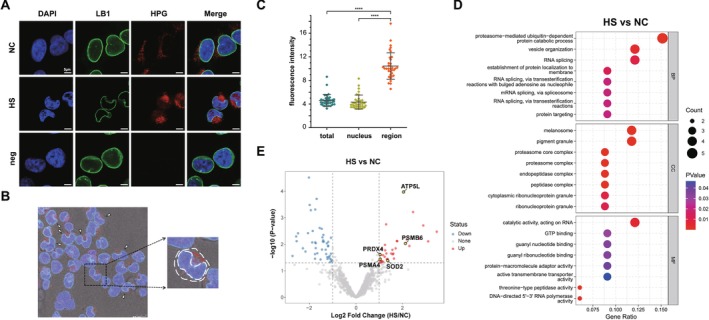
Spatial reprogramming of newly synthesised protein synthesis under heat stress. (A) Spatiotemporal mapping of newly synthesised proteins in heat‐stressed HEK293T cells (HS, 43°C for 1 h) using homopropargylglycine (HPG) pulse labeling. Subsequent click chemistry reaction with Apollo643 azide (red) visualises translation hotspots in nuclear invagination regions. Cells were co‐stained with Lamin B1 (LB1, nuclear membrane, green) and DAPI (nuclei, blue). Negative control (neg) shows complete absence of signal in parallel experiments without HPG pretreatment. Scale bars: 5 μm. (B) Schematic illustration of quantitative analysis of HPG fluorescence enrichment in nuclear invagination regions. White arrows demarcate cells fulfilling nuclear invagination (left). The magnified panel demonstrates the segmentation of nuclear invagination regions (right). (C) Quantification of HPG fluorescence intensity, demonstrating newly synthesised protein enrichment in nuclear invagination regions (*n* = 35). (D) Gene Ontology (GO) term enrichment analysis of newly synthesised proteins from invagination regions. (E) Volcano plot of the newly synthesised proteome under heat stress. The plot displays the statistical significance versus the magnitude of change for proteins identified in the HPG‐based pulldown from heat‐stressed (43°C, 1 h) versus control (NC) cells. Statistically significant proteins (*p* < 0.05) are highlighted, with upregulated proteins (log_2_ FC > 1) shown in red. Key upregulated proteins are explicitly labelled. Statistical analysis: Data in C were analysed using one‐way ANOVA followed by Tukey's post hoc test for multiple comparisons. The significance labels are defined in the Figure 1 legend.

The specific accumulation of organelles within nuclear invagination regions under heat stress suggests a specialised functional role for these compartments. Based on the spatial concentration of organelles in nuclear invagination regions, we investigated the possibility that nuclear invaginations might function as localised hubs for protein quality control, where newly synthesised proteins are processed, despite the overall suppression of global protein synthesis [63, 64]. To assess localised protein synthesis, we used the methionine analog HPG (L‐homopropargylglycine) to label nascent polypeptides, followed by fluorophore conjugation via click chemistry (Figure 3A). Quantitative analysis of HPG fluorescence, comparing invagination regions to the rest of the cell, revealed significantly higher signals (Figure 3B,C), demonstrating a pronounced accumulation of newly synthesised proteins within these compartments. Intriguingly, despite the marked accumulation of newly synthesised proteins, immunofluorescence staining revealed no concomitant enrichment of ribosomal subunits (RPS3, RPL28) or the translation initiation factor eIF2α within the invagination regions (Figure S4A–C). This conclusion was further supported by live‐cell imaging using the SunTag system to visualise active translation sites [65], which also showed no specific enrichment of translation foci within invaginations (Figure S4D). The spatial uncoupling between nascent polypeptides and the core translation machinery suggests that the local HPG enrichment is unlikely to be driven primarily by enhanced local translation efficiency. This finding is more consistent with a scenario in which newly synthesised proteins are recruited to or retained within these compartments after their synthesis elsewhere in the cytoplasm.

To characterise the repertoire of newly synthesised proteins in nuclear invagination regions, we conjugated HPG to biotin tags via click chemistry, followed by streptavidin enrichment and mass spectrometry analysis [66]. Quantitative proteomics analysis identified approximately 1400 proteins on average in the experimental groups. Principal component analysis (PCA) and heatmap correlation analysis demonstrated clear separation of experimental groups and high reproducibility among biological replicates (Figure S4E–H). Western blot confirmed successful biotin conjugation and streptavidin enrichment (Figure S4I,J). Strikingly, Gene Ontology (GO) enrichment analysis of newly synthesised proteins from invagination regions revealed a significant overrepresentation of proteins involved in proteasome‐mediated protein degradation pathways (Figure 3D). Consistently, the volcano plot of the upregulated newly synthesised proteins identified multiple core proteasome subunits, such as PSMA4 and PSMB6 (Figure 3E). These results indicate that heat stress triggers a spatial remodelling of protein metabolism, establishing the invagination region as a degradation hotspot where the newly synthesised proteins actively participate in the regulatory feedback loop that dictates this metabolic reorganisation.

In addition, the upregulated newly synthesised proteins included enzymes critically involved in energy metabolism and reactive oxygen species (ROS) scavenging, such as ATP synthase (ATP5L), superoxide dismutase (SOD2) and peroxidase (PRDX4) (Figure 3E). We sought to validate the spatial recruitment of these components by immunofluorescence. While we confirmed a notable accumulation of ATP5L and PRDX4 within invaginations (Figure S4K,L), we did not observe a similarly pronounced localization for PSMB6 under our imaging conditions (Figure S4M), which may reflect technical or dynamic biological differences. Collectively, this result may reflect a coordinated functional adaptation within the invagination compartment to manage proteotoxic stress during heat stress.

### 2.5 Nuclear Invagination Regions Exhibit Increased Degradation Demands

**FIGURE 4:**
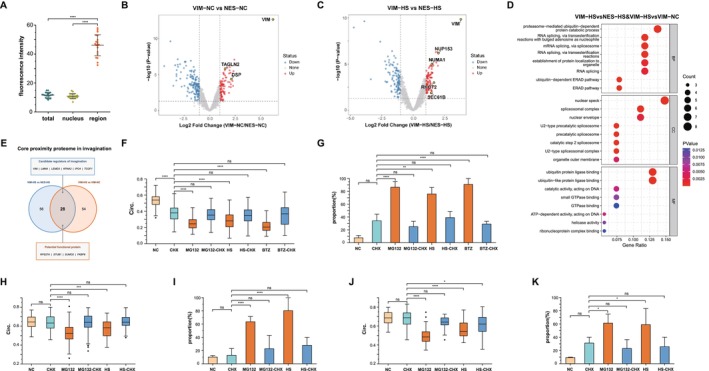
Nuclear invaginations serve as degradation hotspots and are dynamically modulated by cellular degradation demands. (A) Quantification of lysosomal fluorescence intensity, showing enrichment in nuclear invagination regions. (*n* = 20). (B) Volcano plot of the VIM‐proximal proteome under basal conditions. The plot displays the statistical significance versus the magnitude of change for proteins enriched in cells expressing VIM‐APEX2 compared to those expressing NES‐APEX2, both at 37°C (VIM‐NC vs. NES‐NC). Statistically significant proteins (*p* < 0.05) are highlighted, with proteins proximal to VIM (log_2_ FC > 1) shown in red. Key upregulated proteins are explicitly labeled. (C) Volcano plot of the VIM‐proximal proteome under heat stress. The plot displays the statistical significance versus the magnitude of change for proteins enriched in cells expressing VIM‐APEX2 compared to those expressing NES‐APEX2, both under heat stress (43°C, 1 h; VIM‐HS vs. NES‐HS). Statistically significant proteins (*p* < 0.05) are highlighted, with proteins proximal to VIM during heat stress (log_2_ FC > 1) shown in red. Key upregulated proteins are explicitly labeled. (D) Gene Ontology (GO) term enrichment analysis of proximal proteins in nuclear invagination regions. (E) Proximity proteome of the nuclear invagination compartment. Venn diagram identifies proteins significantly enriched in both the VIM‐HS versus NES‐HS and VIM‐HS versus VIM‐NC comparisons, defining high‐confidence components within invaginations. Selected proteins from the intersection are shown, including candidate regulators and potential functional proteins. (F, G) Quantification of nuclear morphology under different pharmacological perturbations shown in Figure S5J. (F) Nuclear circularity. (G) Invagination frequency. (*n*_NC_ = 73, *n*_CHX_ = 99, *n*_MG132_ = 69, *n*_MG132‐CHX_ = 76, *n*_HS_ = 94, *n*_HS‐CHX_ = 91, *n*_BTZ_ = 62, *n*_BTZ‐CHX_ = 67). (H, I) Quantification of nuclear morphology in HeLa cells under different pharmacological perturbations shown in Figure S5K. (H) Nuclear circularity. (I) Invagination frequency. (*n*_NC_ = 51, *n*_CHX_ = 60, *n*_MG132_ = 50, *n*_MG132‐CHX_ = 40, *n*_HS_ = 49, *n*_HS‐CHX_ = 55). (J, K) Quantification of nuclear morphology in Huh7 cells under different pharmacological perturbations shown in Figure S5L. (J) Nuclear circularity. (K) Invagination frequency. (*n*_NC_ = 52, *n*_CHX_ = 26, *n*_MG132_ = 38, *n*_MG132‐CHX_ = 33, *n*_HS_ = 49, *n*_HS‐CHX_ = 58). Statistical analysis: Data in (A, F–K) were analysed using one‐way ANOVA followed by Tukey's post hoc test for multiple comparisons. The significance labels are defined in the Figure 1 legend.

Given that heat stress induces protein denaturation and misfolding [67, 68] and our finding that nuclear invagination regions accumulate newly synthesised proteins, including those involved in degradation pathways, we hypothesised that these regions also experience increased degradation demands during heat stress. Supporting this, live‐cell imaging using the SunTag system frequently revealed bright, aggregated signals within invaginations, indicative of accumulated misfolded or aggregation‐prone polypeptides (Figure S4D). This visual evidence of potential proteostatic burden, coupled with the proteomic enrichment of degradation machinery, pointed to a localised need for clearance. Consistent with this, we also observed lysosomal accumulation within these compartments by immunofluorescence microscopy (Figure 4A; Figure S5A), further suggesting elevated degradation activity.

To directly probe the molecular environment and functional specialisation of nuclear invagination regions, we employed APEX2‐mediated proximity labeling [69]. Using vimentin as a spatial marker for nuclear invagination regions under heat stress, we expressed VIM‐APEX2 in the experimental group and NES‐APEX2 in the control group, conducting labeling at both 37°C and 43°C (Figure S5B). Quantitative mass spectrometry analysis of APEX2‐labelled proteins identified approximately 1000 proteins on average, with PCA and heatmap correlation analysis demonstrating robust separation of experimental groups and high reproducibility of biological replicates. The efficiency of proximity labeling and streptavidin enrichment was validated by Western blot (Figure S5C–G). The specificity of the VIM‐APEX2 proximity labeling was validated by the marked enrichment of VIM itself and its known interaction partners (e.g., DSP, TAGLN2) in the control comparison (VIM‐NC vs. NES‐NC) (Figure 4B). Strikingly, under heat stress (VIM‐HS vs. NES‐HS), the proximal proteome shifted dramatically. We observed a gain of proteins associated with organelles (e.g., AKAP1, RHOT2, SEC61B) and the nuclear envelope (e.g., NUP153, NUMA1) (Figure 4C). This heat stress‐induced remodelling of the VIM‐proximal proteome aligns with our microscopic observations of intermediate filament collapse and perinuclear organelle clustering. Moreover, these dynamic changes also confirm the utility of VIM as a spatially specific marker for targeting the invagination region under heat stress.

Consistent with our hypothesis, GO enrichment analysis of the proximity proteome from heat‐stressed cells revealed a significant overrepresentation of ubiquitin‐ and proteasome‐dependent degradation pathways (Figure 4D). Beyond the proteasomal degradation pathway, our proximity proteome analysis yielded a candidate list of proteins whose localization near the invaginated nuclear envelope suggested potential roles in its formation or regulation. These included factors linked to nuclear structure (LMNA, LEMD3), nucleocytoplasmic transport (KPNA2, IPO4) and ribosome biogenesis (TCOF1) (Figure 4E). However, targeted knockout of these individual candidates did not impair nuclear invagination formation, reinforcing the conclusion that the phenotype is resilient to perturbation of single factors. Furthermore, the proximity proteome also contained proteins involved in ubiquitination (RPS27A, STUB1) and sumoylation (SUMO2) (Figure 4E), indicating that the invagination region is equipped with a more comprehensive protein quality control system. To further validate the increased degradation activity in nuclear invagination regions, we examined ubiquitin accumulation by immunofluorescence. Consistent with our hypothesis, we observed a marked enrichment of ubiquitin conjugates within nuclear invaginations upon heat stress (Figure S5H).

To further confirm that nuclear invagination regions have higher degradation demands, we exposed cells to the proteasome inhibitor MG132 to block basal protein degradation, thereby increasing cellular degradation demands. This treatment induced nuclear invagination phenotypes and significantly reduced nuclear circularity (Figure 4F,G; Figure S5J), accompanied by the accumulation of organelles, such as mitochondria, at the cellular level (Figure S5I). Conversely, if increased protein degradation demand drives nuclear invagination, reducing this demand should prevent or reverse invagination. To test this, we used the translation inhibitor cycloheximide (CHX) to suppress global protein synthesis, thereby reducing the load of proteins requiring degradation. Indeed, CHX treatment rescued the nuclear invagination phenotype induced by MG132 (Figure 4F,G; Figure S5J). Furthermore, CHX treatment also prevented the appearance of nuclear invaginations under heat stress (Figure 4F,G; Figure S5J). Notably, the induction of invaginations by proteasome inhibition (MG132) and their suppression by translational inhibition (CHX) were consistently observed across diverse cell types, including HeLa and Huh7 (Figure 4H–K; Figure S4K,L). Besides, this core regulatory relationship was confirmed using an alternative, highly specific proteasome inhibitor, bortezomib (BTZ) (Figure 4F,G; Figure S5J). The consistent response underscores that the coupling between protein degradation demand and nuclear morphology is a fundamental cellular property, rather than an artefact of a specific drug or cell line. Collectively, these experimental results indicate that nuclear invagination regions exhibit increased degradation demands and nuclear invagination can be modulated by altering cellular degradation demands.

### 2.6 Nuclear Invaginations Facilitate Cellular Heat Stress Response

**FIGURE 5:**
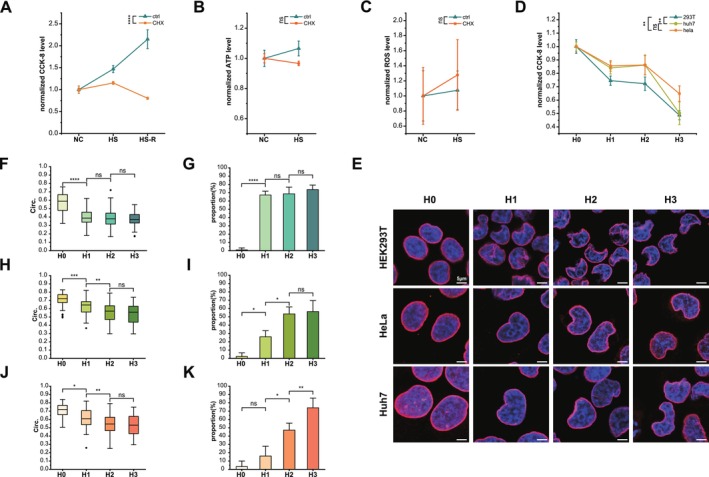
Nuclear invaginations sustain cell viability under heat stress and correlate with cellular heat tolerance. (A–C) Cell viability assays analysing the effect of Cycloheximide (CHX, 100 μg/mL for 20 h) inhibition of nuclear invagination formation during subsequent heat stress (HS, 43°C for 1 h) compared to control (ctrl, DMSO). (A) CCK‐8 assay. (B) ATP level detection assay. (C) Reactive Oxygen Species (ROS) level detection assay. (D) CCK‐8 assays of three different cell lines (HEK293T, HeLa and Huh7) subjected to increasing heat stress durations (43°C; 1 h, 2 h, 3 h treatment). (E) Immunofluorescence (stained with Lamin B1 and DAPI) showing nuclear morphology alterations in HEK293T, HeLa and Huh7 cells under different heat stress duration gradients (43°C; 1 h, 2 h, 3 h treatment). Scale bars: 5 μm. (F–K). Quantification of nuclear morphology in the three cell lines under heat stress duration gradients (43°C; 1 h, 2 h, 3 h treatment). (F, H, J) represent nuclear circularity for HEK293T, HeLa and Huh7 cells, respectively. (G, I, K) represent invagination frequency for HEK293T, HeLa and Huh7 cells, respectively. (For (F, G), *n*_H0_ = 82, *n*_H1_ = 89, *n*_H2_ = 101, *n*_H3_ = 93. For (H, I), *n*_H0_ = 40, *n*_H1_ = 66, *n*_H2_ = 57, *n*_H3_ = 60. For (J, K), *n*_H0_ = 25, *n*_H1_ = 48, *n*_H2_ = 64, *n*_H3_ = 35). Statistical analysis: Data in (A–D) were analysed using Two‐way ANOVA followed by Tukey's post hoc test for multiple comparisons. Data in (F–K) were analysed using One‐way ANOVA followed by Tukey's post hoc test for multiple comparisons. The significance labels are defined in the Figure 1 legend.

Having established nuclear invagination regions as metabolic hotspots with enhanced degradation, we next investigated their functional role in cellular heat stress response. We hypothesise that nuclear invaginations represent a novel regulatory mechanism for cellular adaptation to heat stress. To test this, we used CHX to inhibit nuclear invaginations formation during heat stress. Considering its role as a translation inhibitor and its potential negative impact on cell viability, experimental groups were normalised to CHX‐treated controls to account for the confounding effects of translation inhibition. CCK‐8 assays demonstrated that cells lacking nuclear invaginations exhibited reduced survival rates both during heat stress and after 24 h of recovery compared to control cells (Figure 5A), indicating that nuclear invaginations are critical for both heat stress tolerance and subsequent recovery.

Having established that nuclear invaginations promote cell survival under heat stress, we sought to explore potential underlying mechanisms. Given the proteomic data of newly synthesised proteins enrichment of energy metabolism and antioxidant components in these regions, we hypothesised that invaginations might help maintain metabolic and redox homeostasis. To test this, we assayed cellular ATP levels and ROS levels under heat stress. Consistent with our hypothesis, CHX‐treated cells lacking nuclear invaginations showed a non‐significant trend toward lower cellular ATP levels (*p* = 0.056, Figure 5B) and a concomitant trend toward elevated ROS (*p* = 0.872, Figure 5C). Although these global measurements did not reach statistical significance, the co‐directional shifts align with the proteomic signature and the observed survival deficit. This consistency suggests that the spatial compartmentalization of stress‐relevant machinery within nuclear invaginations may serve to buffer local metabolic demands and mitigate oxidative damage, potentially contributing to overall cellular adaptation. Thus, the nuclear invagination likely represents a novel phenotypic regulatory mechanism, as indicated by the convergence of proteomic, imaging and functional observations.

### 2.7 Nuclear Invaginations Correlate With Heat Tolerance Across Cell Lines

Having demonstrated the functional importance of nuclear invaginations in cellular heat shock response, we next investigated whether the capacity to form these structures correlates with intrinsic heat tolerance across different cell lines. To determine the relative heat tolerance of different cell lines, we first assessed cell viability in HEK293T, HeLa and Huh7 cells across a time course of heat shock using CCK‐8 assays (Figure 5D). These assays revealed a clear hierarchy of heat tolerance: HeLa cells exhibited the highest viability, followed by Huh7, whereas HEK293T cells were the most susceptible to heat stress. Specifically, during the first 2 h of heat shock, HeLa and Huh7 cells exhibited higher viability than HEK293T cells, indicating that HEK293T cells were the most susceptible to short‐term heat stress. Between 2 and 3 h of heat exposure, HeLa cells maintained the highest viability, whereas Huh7 cells showed a marked decrease in viability, suggesting reduced tolerance to prolonged heat stress (Figure 5D).

We next investigated whether the capacity to form nuclear invaginations also varied across these cell lines and if so, how it related to their heat tolerance levels. We quantified nuclear invagination formation in these three cell lines at different time points. HEK293T cells showed the highest capacity for nuclear invagination formation (Figure 5E), manifested by both the highest percentage of cells with nuclear invaginations and the largest changes in nuclear circularity (Figure 5F,G). In contrast, HeLa and Huh7 cells developed pronounced nuclear invagination phenotypes only after longer heat shock exposure (Figure 5E) and exhibited lower overall levels of invagination compared to HEK293T cells (Figure 5H–K). These results demonstrate that different cell lines exhibit distinct capacities to form nuclear invaginations for heat stress tolerance, with lower tolerant cells showing enhanced nuclear invagination formation to facilitate their stress response.

## 3 Discussion

Our study unveils a previously unrecognised cellular response to heat stress: the formation of reversible nuclear invaginations that establish spatially organised hubs for protein metabolism. These invaginations, characterised by nuclear shrinkage, kidney‐shaped morphology, perinuclear intermediate filament collapse and accumulation of mitochondria and lysosomes, are not merely morphological aberrations, but rather represent a novel subcellular compartment dedicated to coordinating essential stress response processes. Specifically, we demonstrate that they concentrate newly synthesised proteins alongside degradation machinery, suggesting they may function as specialised hubs for coordinated protein processing during stress. Integrated imaging and proteomic analyses revealed a selective enrichment of proteins involved in proteostasis, including both degradation machinery and components for energy and redox homeostasis. This unique composition positions nuclear invaginations as potential sites for the coordinated management of protein metabolism during stress.

It is worth considering why such a frequent and prominent phenomenon as heat‐induced nuclear invagination has not been extensively documented in previous studies. We speculate that this oversight may stem from a combination of methodological constraints and inherent observer bias. Traditionally, nuclear morphology is assessed using DNA dyes like DAPI or Hoechst, which effectively visualise chromatin but fail to precisely delineate the physical boundaries of the nuclear envelope. Due to chromatin heterogeneity and the constraints of single‐focal‐plane imaging, nuclear invaginations under DAPI staining alone are easily misinterpreted as uneven chromatin distribution or localised signal loss. In contrast, our dual staining with Lamin B1 antibodies and DAPI provides a continuous, high‐definition outline of the nuclear membrane, enabling the precise identification of invaginations that remain ‘invisible’ or ambiguous under conventional DNA staining. Furthermore, during microscopic observation and image presentation, researchers often exhibit a preference for ‘representative’ cells with symmetric, elliptical nuclei. Consequently, stress‐induced invaginations have likely been dismissed as preparation artefacts or signs of poor cell health rather than biologically significant responses. By integrating systematic quantification with multidimensional imaging, our study recontextualizes these deformations as regulated cellular processes, prompting a re‐examination of this long‐neglected morphological feature.

Our systematic search for essential upstream regulators of heat stress‐induced nuclear invagination yielded a notable result: under our experimental conditions, we were unable to identify a single, non‐redundant gene or a canonical signalling pathway whose perturbation reproducibly abolished nuclear invagination. Although this does not rule out the involvement of specific factors that our screening approach might have missed, it suggests that the phenotype is not strictly dependent on any individual component we tested. This could indicate that heat stress‐induced nuclear invagination is a complex and potentially redundant cellular response, making it inherently resistant to disruption by targeting single nodes. Consequently, the formation of this structure may be better understood as an emergent property arising from integrated changes in global cellular states—such as proteostatic stress and cytoskeletal reorganisation—rather than being triggered by a linear, dedicated pathway. This insight led us to reconsider our approach: Instead of continuing to search for a unique upstream trigger, we turned to investigating the functional output and metabolic logic of the invagination compartment itself, reasoning that understanding its utility to the cell might provide more fundamental clues about its regulation.

Our subsequent analysis of the compartment's function reveals it as a spatially organised hub for protein metabolism. During heat stress, despite global transcriptional and translational suppression, cells must manage the conflicting demands of producing essential stress proteins while mitigating widespread protein damage. To adapt to this challenge, cells undergo spatial reorganisation to form specialised nuclear invaginations, representing a strategic redistribution of cellular resources and energy. This compartmentalization may serve to concentrate newly synthesised proteins and the machinery for their processing and degradation. By locally enriching factors such as chaperones, proteasome components and energy supply molecules within invagination regions, cells could prioritise the folding, quality control and turnover of critical polypeptides even under conditions of global translational inhibition. Heat stress also induces misfolding of nascent proteins and general protein denaturation. Rapid clearance of these aberrant proteins constitutes a critical component of the cellular stress response.

We propose that nuclear invagination regions serve as degradation hotspots, alleviating the burden of misfolded protein accumulation, reducing secondary damage caused by denatured proteins and preserving cellular homeostasis. The co‐accumulation of newly synthesised proteins and active degradation machinery within the same compartment suggests a tightly coupled system for protein quality control. This spatial coupling could create a local feedback loop: The recruitment of newly synthesised stress‐response factors is matched by an immediate degradation capacity, ensuring rapid clearance of misfolded intermediates. This would maintain a protected proteostatic environment within the invagination and prevent proteotoxic damage from spreading to the wider cytoplasm. Nevertheless, the precise molecular mechanisms that initiate this compartmentalization and coordinate its functions remain to be fully elucidated. Future research should focus on identifying the upstream triggers for invagination, the signals regulating its dynamics and the principles governing the specific recruitment and retention of protein quality control components within these regions. Furthermore, defining the proteomic composition of invagination regions in more detail will be crucial for understanding their functional specialisation.

An intriguing aspect of our findings is the functional composition of the invagination compartment. Despite its role as a protein metabolism hotspot, our newly synthesised proteome analysis did not reveal significant enrichment of classical HSPs. This temporal dissociation is critical. Consistent with the established kinetics of the HSR [17, 70], our whole‐cell proteomic time course revealed that pathways related to protein folding and the heat shock response (Figure S6A), as well as the expression of core HSP machinery and specific chaperones (Figure S6B,C), showed only modest induction during the acute 1‐h heat stress (H1). Strikingly, their pronounced upregulation became evident only during the subsequent recovery phase (H1R6 onwards). This pattern underscores the inherent delay between HSR transcriptional activation and substantial protein accumulation. Therefore, our results position nuclear invagination as a rapid, subcellularly organised coping strategy that operates in parallel to—and importantly, precedes—the full‐scale transcriptional‐protein synthetic wave of the HSR. We propose that by spatially concentrating machinery for nascent protein processing, degradation, energy supply and antioxidant defence, this compartment serves as an immediate frontline system to manage proteotoxic stress. It thereby bridges the critical proteostatic gap before the delayed, cell‐wide chaperone response is fully deployed hours later, representing a novel layer of cellular adaptation to acute thermal stress.

Our findings reveal that nuclear invagination is a cell type–dependent adaptation to heat stress, with its extent correlating with intrinsic heat tolerance: the less tolerant HEK293T cells form more invaginations, whereas the more tolerant HeLa cells form fewer. This suggests that invaginations may represent a compensatory or protective response that is more critical for cells with inherently weaker stress defences. Two factors may explain this divergence. First, variations in basal biochemical defences may dictate the degree of reliance on inducible responses. As typical cancer cells, HeLa and Huh7 exhibit pronounced ‘chaperone addiction’ with high basal expression of HSPs [71, 72]. This robust pre‐existing defence likely enables them to maintain systemic protein homeostasis effectively under acute heat stress, potentially resulting in higher heat tolerance and a reduced reliance on inducible mechanisms such as nuclear invaginations. In contrast, HEK293T cells, which are characterised by intensive protein synthesis and fragile basal defences [73], face protein folding pressures that may exceed their biochemical buffering limits, plausibly necessitating greater reliance on inducible responses like nuclear invagination. Second, nuclear mechanical properties may set a structural threshold for invagination. Data from HPA reveal that epithelial‐derived HeLa cells highly express lamin A/C and vimentin [74, 75]. Lamin A/C is thought to impart higher mechanical stiffness to the nucleus [76], whereas the abundant vimentin network might provide enhanced cytoskeletal support during heat stress [77], potentially raising the barrier to deformation. Conversely, HEK293T cells express lower levels of these proteins, resulting in a more mechanically plastic nucleus that may facilitate invagination. Future research is warranted to validate the nuclear invagination phenotype in more physiologically relevant models, such as primary cells. This includes determining whether the mechanism of degradation‐demand‐induced invagination is universal, identifying the factors that dictate differences in invagination‐forming capacity across various cell types and investigating the evolutionary conservation of this phenotype across diverse species. Such explorations will further elucidate the fundamental biological significance of nuclear invaginations and deepen our understanding of the intricate relationships between nuclear structural dynamics, proteostasis and cellular stress responses.

Heat stress–induced nuclear invagination morphologically resembles the nuclear envelope invagination or dysmorphism observed in pathologies such as cancer, aging and cardiomyopathy [47, 48, 49, 50]. However, these two phenomena differ significantly in their inducers and dynamics. Heat stress‐induced nuclear invagination is an actively activated, rapid adaptation to restore cellular homeostasis, concentrating protein turnover resources. Crucially, it is highly reversible upon stimulus removal. Conversely, disease‐related invaginations are considered structural aberrations resulting from long‐term damage or pathological stimuli, persisting continuously and correlating with disease progression. Our findings suggest a potential hypothesis: Pathological nuclear envelope reshaping may reflect a dysregulated metabolic remodelling process. Much like the ubiquitous protein homeostasis imbalance found in cancer and neurodegenerative diseases, we propose that nuclear envelope abnormalities influenced by pathological factors might trigger a protein metabolic remodelling process similar to that seen during heat stress. However, these pathological cells lack the precise, controllable mechanisms to effectively regulate the invagination. This leads to the loss of control over the remodelling process, consequently causing further imbalance in protein homeostasis and accelerating the onset and progression of the disease. Studying the controllable, adaptive nuclear invagination during heat stress can deepen our understanding of the pathogenic mechanisms of nuclear envelope abnormality‐related diseases at the protein metabolism level. This may offer new directions for developing structure‐remodelling‐based therapeutic strategies.

## 4 Limitations

Our study provides initial evidence identifying the unique phenomenon of heat stress‐induced nuclear invagination and characterising this region as a ‘protein metabolism hotspot’ during stress, thereby contributing to the understanding of cellular stress adaptation mechanisms. While these findings open a novel avenue for research, our work still presents certain limitations, which simultaneously point toward promising directions for future investigation.

Through high‐resolution imaging, we observed that nuclear invagination formation is closely associated with the collapse of the intermediate filament network and the regional clustering of specific organelles (e.g., mitochondria and lysosomes) near the nuclear envelope. However, the precise causal chain among these observed phenotypic changes and the core driving force behind nuclear invagination remain to be fully elucidated. We attempted to interfere with several potential upstream regulators, including heat shock proteins, nuclear envelope components and cytoskeletal genes. Yet, potentially due to complex multi‐gene redundancy, cellular compensatory mechanisms, or inherent difficulties in achieving sufficient knockdown efficiency of essential genes, we were unable to pinpoint a definitive ‘molecular switch’ that directly governs nuclear invagination formation. Therefore, future efforts will be essential to explore the upstream signalling pathway or core effector molecule that directly initiates nuclear membrane remodelling and nuclear invagination formation under heat stress, utilising more refined genetic screening, functional imaging and biochemical analysis approaches, to more comprehensively uncover the mechanism underlying this subcellular reorganisation.

Furthermore, our functional analyses were predominantly carried out using chemical inhibitors. We employed the proteasome inhibitor MG132 and the translation inhibitor CHX to artificially manipulate nuclear invagination formation, subsequently assessing its protective role in cellular heat stress response and survival. This core regulatory logic was consistently observed across multiple cell types (HeLa, Huh7) and with an alternative proteasome inhibitor (BTZ). These experiments provided initial correlational evidence suggesting the importance of nuclear invagination as a protein translation and degradation hotspot. Nevertheless, it must be acknowledged that both MG132/BTZ and CHX are broad‐spectrum inhibitors and their pleiotropic effects are far from specific to nuclear invagination itself. Their widespread effects on global translation, protein degradation and various signalling pathways potentially introduce confounds when attempting to achieve the independent assessment of nuclear invagination function. Moving forward, the successful identification of the specific upstream molecules or effectors that regulate nuclear invagination formation will allow for the development of more specific genetic or pharmacological tools. This will enable the precise, independent manipulation of nuclear invagination, which is crucial for a more comprehensive and rigorous unveiling of the distinct physiological role nuclear invagination plays in cellular stress adaptation.

## 5 Methods

### 5.1 Cell Culture

HEK293T, Huh7 and HeLa cells were maintained in Dulbecco's Modified Eagle Medium (DMEM; Corning) supplemented with 10% (v/v) fetal bovine serum (FBS; Gibco) at 37°C in a humidified atmosphere containing 5% CO_2_. All cell lines were routinely tested for mycoplasma and confirmed to be negative. Heat shock treatments were performed using a temperature‐controlled water bath or cell culture incubator.

### 5.2 Plasmid Construction and Cell Line Generation

For proximity labeling experiments, VIM and NES gene fragments were PCR‐amplified from cDNA templates, whereas the APEX2 gene fragment was PCR‐amplified from a template plasmid (Addgene #124617). These fragments were inserted into the plx304 backbone (Addgene #124617) using seamless cloning. The resulting constructs were transformed into competent *E. coli* cells using a standard heat shock protocol and positive colonies were selected on LB‐ampicillin plates. All plasmid constructs were verified by Sanger sequencing.

To establish VIM‐APEX2 and NES‐APEX2 stable cell lines, the corresponding plasmids were co‐transfected with lentiviral packaging plasmids into HEK293T cells using Lipofectamine 2000 (Invitrogen, cat. no. 11668019). Viral supernatants were harvested 48 h post‐transfection, centrifuged and filtered through a 0.45 μm filter. HEK293T cells were then transduced with the filtered viral supernatants and selected with 8 μg/mL blasticidin (MCE, cat. no. HY‐K1054).

For LMNB1 fluorescent reporter cell line generation, we followed the protocol described previously [78]. Briefly, a BSD::P2A::mCherry cassette was inserted upstream of the endogenous LMNB1 gene using CRISPR/Cas9‐mediated gene editing. The sgRNA sequence targeting LMNB1 (5′‐GGGGTCGCAGTCGCCATGGC‐3′) was designed based on the previous study. The sgRNA construct, Cas9 expression plasmid and donor repair template were co‐transfected into cells using Lipofectamine 2000. Stable integrants were selected with 8 μg/mL blasticidin.

### 5.3 Genetic Perturbation and Candidate Gene Screening

For targeted genetic screening, candidate genes spanning nuclear integrity, cytoskeletal dynamics and stress signalling pathways were perturbed using either CRISPR‐Cas9‐mediated knockdown or plasmid‐based overexpression.

For CRISPR‐Cas9 knockdown, the sgRNAs targeting each candidate gene were designed using CRISPick and cloned into the lentiCRISPR v2 backbone. The sgRNA sequences are provided in Table S1. Cells were co‐transfected with the sgRNA plasmid and a Cas9 expression plasmid using Lipofectamine 2000. Transfected cells were treated with puromycin for 48 h and typically analysed 48–72 h post‐transfection.

For overexpression, open reading frames of candidate genes were PCR‐amplified and cloned into expression vector pcDNA3.1. Cells were transiently transfected with these plasmids using Lipofectamine 2000. Transfected cells were treated with puromycin for 48 h and typically analysed 48–72 h post‐transfection.

Knockdown and overexpression efficiency was validated by RT‐qPCR using HiScript II One Step qRT‐PCR SYBR Green Kit (Vazyme, cat. no. Q221‐01). The relative expression in knockdown cells was calculated using the 2^−ΔΔCt^ method compared to wild‐type (WT) controls.

### 5.4 Immunofluorescence

Cells were seeded in glass‐bottom dishes and cultured until appropriate confluence. During the experiment, the culture medium was aspirated and cells were washed with PBS. Cells were fixed with 4% paraformaldehyde for 15 min at 4°C, permeabilized with pre‐chilled methanol for 10 min at −20°C and blocked with 6% (v/v) goat serum (Bioss) in PBS for 1 h at 4°C. Cells then sequentially incubated with primary antibodies, fluorophore‐conjugated secondary antibodies and DAPI. Between each step, cells were washed three times with PBS for 5 min each on a shaker. The samples were maintained in PBS for imaging.

The antibodies used in this study were as follows: Mouse anti‐Lamin B1 (Proteintech, cat. no. 66095‐1‐Ig), Rabbit anti‐DDDDK‐Tag monoclonal antibody (Abclonal, cat. no. AE092), Mouse anti‐β‐Tubulin monoclonal antibody (Abclonal, cat. no. AC021), Rabbit anti‐eIF6 polyclonal antibody (Abclonal, cat. no. A1818), Rabbit anti‐G3BP1 monoclonal antibody (Abclonal, cat. no. A3968), Rabbit anti‐β‐Actin monoclonal antibody (Abclonal, cat. no. A2319), Rabbit anti‐Vimentin monoclonal antibody (Abclonal, cat. no. A19607), Rabbit anti‐LAMP1 monoclonal antibody (Abclonal, cat. no. A23947), Rabbit anti‐Nesprin1 (SYNE1) polyclonal antibody (Abclonal, cat. no. A20256), Rabbit anti‐NUP153 monoclonal antibody (Abclonal, cat. no. A2472), Rabbit anti‐PSMB6 polyclonal antibody (Abclonal, cat. no. A4053), Rabbit anti‐ATP5L polyclonal antibody (Abclonal, cat. no. A9178), Rabbit anti‐PRDX4 polyclonal antibody (Abclonal, cat. no. A18308), Rabbit anti‐SUN1 monoclonal antibody (Zenbio, cat. no. R383167), Rabbit anti‐eIF2A monoclonal antibody (Zenbio, cat. no. R24185), Goat anti‐mouse IgG (H + L) conjugated to ABflo488 (Abclonal, cat. no. AS037), Goat anti‐rabbit IgG (H + L) conjugated to ABflo594 (Abclonal, cat. no. AS074), Alexa Fluor 594‐conjugated streptavidin (Yeasen, cat. no. 35107ES60).

### 5.5 SunTag Live‐Cell Imaging

As previously described [65], cells were co‐transfected with two plasmids: one expressing a Kif18b protein fused to the 24 × GCN4 SunTag array (Addgene #74928) and another expressing a single‐chain anti‐GCN4 antibody (scFv) fused to superfolder GFP (sfGFP) (Addgene #60907). Forty‐eight hours after transfection, cells were subjected to heat stress at 43°C. Following heat treatment (or normal culture for the NC group), cells were fixed with 4% paraformaldehyde and stained with DAPI to label the nuclear. Imaging was performed on a Leica TCS SP8 STED 3× microscope using standard filter sets for GFP and the corresponding fluorophores.

### 5.6 Microscopy and Image Acquisition

Imaging was performed on a Leica TCS SP8 STED 3X microscope equipped with a 100× oil‐immersion objective. Standard fixed cell imaging was conducted with a minimum of three random fields per condition, whereas three‐dimensional reconstruction was achieved through *z*‐stack acquisition at optimal step sizes and processed using LAS × Office software.

Live‐cell imaging was performed using a Nikon ECLIPSE Ti2‐E spinning disk confocal microscope equipped with a CSU‐W1 confocal unit and a 63×/1.4 NA oil‐immersion objective. Cells were maintained at 37°C in a humidified atmosphere containing 5% CO_2_ using an environmental control chamber.

For transmission electron microscopy (TEM), heat‐shocked cells were harvested by scraping and fixed with 2.5% glutaraldehyde. Sample processing and imaging were performed by Nocen Bio using a JEOL JEM1400 transmission electron microscope.

### 5.7 Statistical Analysis Methods

Confocal microscopy images were analysed using ImageJ (NIH). Nuclear morphology was quantified by measuring cell circularity using the Enclosing plugin on Lamin B1‐stained channels. For protein and organelle accumulation analysis, cells were segmented based on bright‐field and Lamin B1‐stained channel images. Regions of significant membrane invagination were identified and the mean fluorescence intensity of the target was measured within these defined regions. Cells partially outside the field of view or those with poorly defined nuclear boundaries were excluded from analysis. All quantitative data were analysed and visualised using OriginPro (OriginLab).

### 5.8 Apoptosis Analysis

Cell were harvested by trypsinization, pelleted by centrifugation and washed with ice‐cold PBS. Apoptosis was assessed using an Annexin V‐FITC/PI Apoptosis Detection Kit (Dojindo, cat. no. AD10) according to the manufacturer's instructions. After staining, samples were analysed using a CytoFLEX flow cytometer (Beckman Coulter). A minimum of 10,000 events were collected per sample and data analysis was performed using FlowJo VX (BD Biosciences) to quantify the proportions of viable, early apoptotic, late apoptotic and necrotic cells.

### 5.9 Western Blot Analysis

Total protein was extracted using 6× SDS loading buffer (Beyotime, cat. no. P0015F). Samples were denatured at 100°C for 15 min, separated by 10% SDS‐PAGE, and transferred to PVDF membranes (Millipore, cat. no. ISEQ00010). Membranes were blocked with 5% non‐fat milk in TBST for 1 h at room temperature, followed by overnight incubation with primary antibodies at 4°C. After washing five times with TBST for 5 min each, membranes were incubated with secondary antibodies for 2 h at room temperature. Following five additional TBST washes, protein bands were visualised using ECL substrate (Bio‐Rad, cat. no. 1705061) and detected using a Tanon chemiluminescence imaging system.

For proteomic samples, 5% of the total volume was retained during enrichment and processed as described above. For biotinylation analysis, HRP‐conjugated streptavidin (Proteintech, cat. no. SA00001) was used in place of primary and secondary antibodies.

### 5.10 Proximity Labeling Experiment

Proximity labeling was performed as previously described [69]. Briefly, cells were incubated with 500 μM biotin‐phenol (BP) in complete medium for 30 min at 37°C. Biotinylation was initiated by adding H_2_O_2_ to a final concentration of 1 mM and terminated after 1 min by aspirating the medium and washing cells three times with quenching buffer (10 mM sodium ascorbate, 5 mM Trolox and 10 mM sodium azide in DPBS). Cells were lysed in RIPA buffer (Beyotime, cat. no. P0013K) supplemented with protease inhibitor cocktail, followed by sonication. Biotinylated proteins were enriched using Streptavidin Sepharose High Performance beads (Cytiva, cat. no. 17511301) for subsequent proteomic analysis.

### 5.11 Newly Synthesised Protein Detection Experiment

Newly synthesised protein labeling was performed using L‐homopropargylglycine (HPG) incorporation. Specifically, cells were methionine‐depleted in methionine‐free medium (Beyotime, cat. no. C0891) for 24 h, followed by incubation with 100 μM HPG (Beyotime, cat. no. ST2057) for 1 h at 37°C.

For immunofluorescence analysis, HPG‐labelled proteins were conjugated to Apollo643 azide (RiboBio, cat. no. C10316) via copper‐catalysed click reaction, following standard immunofluorescence protocols. Click reaction was performed at 25°C for 2 h under conditions of 1 mM CuSO_4_, 2 mM BTTAA and 10 mM sodium ascorbate.

For proteomic analysis, cells were lysed in RIPA buffer supplemented with protease inhibitors and sonicated. Proteins were purified using Microcon‐30 kDa Centrifugal Filter Units (Merck Millipore, cat. no. MRCF0R030), followed by click reaction with biotin azide (UElandy, cat. no. B5062). The labelled proteins were re‐purified using centrifugal filters and subjected to streptavidin enrichment for subsequent proteomic analysis.

### 5.12 Streptavidin Enrichment and Proteomic Analysis

Biotinylated proteins were enriched using Streptavidin Sepharose High Performance beads. Beads were pre‐washed twice with RIPA buffer, followed by sample incubation at 4°C for 4 h with rotation. Sequential washing steps were performed for 5 min each: 1 M KCl, 0.1 M NaCO3, 2 M Urea in 10 mM Tris–HCl (pH 8.0), RIPA buffer, 50 mM Tris–HCl (pH 7.5) and 2 M Urea in 50 mM Tris–HCl (pH 7.5) (twice). Pre‐digestion was performed in 2 M Urea in 50 mM Tris–HCl (pH 7.5) containing 1 mM DTT and 0.4 μg trypsin at 25°C for 1 h. Supernatant was collected and combined with two additional bead washes. Proteins were reduced with 4 mM DTT at 25°C for 30 min, alkylated with 10 mM IAA at 25°C for 45 min in dark and ingested with 0.5 μg more trypsin at 37°C for 16 h.

Peptides were desalted using Pierce C18 columns (Thermo Scientific, cat. no. 87784) according to the manufacturer's protocol, flash‐frozen in liquid nitrogen and lyophilized (Christ Alpha 3–4 LSD basic). Samples were reconstituted in 0.1% formic acid and analyzed using Q Exactive HF mass spectrometer in data‐dependent acquisition (DDA) mode.

Raw data were processed using MaxQuant software with the following parameters: Database: UniProt canonical human proteome (UP000005640_9606), false discovery rate (FDR): 1%, quantification: Label‐free quantification (LFQ), minimum peptide count for LFQ: 2 and other parameters: Default settings. For proximity labeling analysis, the proteins significantly enriched in both VIM‐HS vs. VIM‐NC and VIM‐HS vs. NES‐HS comparisons were considered as the proteome of nuclear invagination regions. For heat‐induced newly synthesised proteome analysis, the differential proteins between HPG‐HS and HPG‐NC were considered as the newly synthesised proteome upon heat stress.

### 5.13 Cell Viability Assay

Cells were seeded in 96‐well plates and cultured until appropriate confluence before being treated with drugs or heat shock. Cell viability was assessed using Cell Counting Kit‐8 (TargetMol, cat. no. C0005). In brief, CCK‐8 reagent was added at a 1:10 ratio and incubated at 37°C for 1 h. Absorbance was measured at 450 nm using a BioTek microplate reader, with blank controls for correction.

ATP levels were determined using the Enhanced ATP Assay Kit (Beyotime, cat. no. S0027). After removing culture medium, cells were lysed and analysed following the manufacturer's protocol. Luminescence (CPM) was recorded using a BioTek microplate reader, with blank controls for correction.

Reactive oxygen species were measured using DCFH‐DA probe (Beyotime, cat. no. S0033S). In brief, cells were incubated with 10 μM DCFH‐DA in serum‐free medium at 37°C for 20 min, followed by three washes with serum‐free medium. Fluorescence intensity was measured using a BioTek microplate reader (excitation: 488 nm; emission: 525 nm) with blank controls for correction.

### 5.14 Statistics and Reproducibility

Data are presented as mean ± SD. Statistical significance was assessed using one‐way ANOVA with Tukey's post hoc test for comparisons among three or more groups, two‐way ANOVA with Tukey's test for datasets with two independent variables. A P value of less than 0.05 was considered statistically significant and significance levels in figures are denoted as: ns, not significant; *p < 0.05; **p < 0.01; ***p < 0.001; ****p < 0.0001. For immunofluorescence quantification, analyses were performed on a minimum of three independent fields and representative images are shown from independent experiments. Sample sizes (*n*) are indicated in figure legends or on the graphs. For proteomics and cell viability assays, all experiments were performed with three independent biological replicates.

## Author Contributions

G.‐Z.L. conceived the project; Z.‐H.Z., Y.‐F.H., Y.‐T.Q. wrote the manuscript with the help of Y.‐L.L., C.‐L.W.; Z.‐H.Z., Y.‐T.Q., J.‐Y.C. conducted the experiments with the help of Y.‐F.H., Y.‐L.Z., F.W., C.‐L.W., J.‐Y.J.; Y.‐T.Q., Z.‐H.Z. performed the bioinformatics analysis with the help of Y.‐L.L., G.‐R.T.; G.‐Z.L. and Z.Z. supervised the project. All authors participated in the discussion of the project and editing of the manuscript.

## Ethics Statement

The authors have nothing to report.

## Conflicts of Interest

The authors declare no conflicts of interest.

## Supporting information


**Data S1:** Differentially expressed newly synthesised proteins identified by proteome assay.

**Data S2:** Differentially expressed proteins identified by proximity labeling proteome assay.

**Figure S1:** Supplementary figures.

**Table S1:** gRNA sequences for knockout assay.

**Video S1:** Real‐time video for nuclear invagination dynamics under heat stress and recovery. Stress phase: 0–36 min at 43°C. Recovery phase: 37°C for 135 min. CO_2_ maintained at 5% throughout.

## Data Availability

The sequence data generated in this study have been deposited in the NCBI Gene Expression Omnibus (GEO), under accession number GSE292106. The mass spectrometry proteomics data have been deposited to the ProteomeXchange Consortium via the iProX partner repository with the dataset identifier PXD073536. The list of differential proteins identified in this study is provided as Data S1 and S2.

## References

[1] K. L. O'Neill , D. W. Fairbairn , M. J. Smith , and B. S. Poe , “Critical Parameters Influencing Hyperthermia‐Induced Apoptosis in Human Lymphoid Cell Lines,” Apoptosis 3, no. 5 (1998): 369–375. 14646484 10.1023/a:1009689407261

[2] E. Falcieri , F. Luchetti , S. Burattini , B. Canonico , S. Santi , and S. Papa , “Lineage‐Related Sensitivity to Apoptosis in Human Tumor Cells Undergoing Hyperthermia,” Histochemistry and Cell Biology 113, no. 2 (2000): 135–144. 10766266 10.1007/s004180050016

[3] T.‐T. Wang , A. S. Chiang , J. J. Chu , T. J. Cheng , T. M. Chen , and Y. K. Lai , “Concomitant Alterations in Distribution of 70kDa Heat Shock Proteins, Cytoskeleton and Organelles in Heat Shocked 9L Cells,” International Journal of Biochemistry & Cell Biology 30, no. 6 (1998): 745–759. 9695029 10.1016/s1357-2725(97)00133-7

[4] F. Luchetti , F. Mannello , B. Canonico , et al., “Integrin and Cytoskeleton Behaviour in Human Neuroblastoma Cells During Hyperthermia‐Related Apoptosis,” Apoptosis 9, no. 5 (2004): 635–648. 15314292 10.1023/B:APPT.0000038043.03799.6f

[5] R. A. Coss and W. A. M. Linnemans , “The Effects of Hyperthermia on the Cytoskeleton: A Review,” International Journal of Hyperthermia 12, no. 2 (2009): 173–196. 10.3109/02656739609022507 8926388

[6] A. Pawlik , J. M. Nowak , D. Grzanka , L. Gackowska , J. Michalkiewicz , and A. Grzanka , “Hyperthermia Induces Cytoskeletal Alterations and Mitotic Catastrophe in p53‐Deficient H1299 Lung Cancer Cells,” Acta Histochemica 115, no. 1 (2013): 8–15. 22483983 10.1016/j.acthis.2012.02.006

[7] R. A. Kammerer , D. Kostrewa , J. Zurdo , et al., “Exploring Amyloid Formation by a De Novo Design,” Proceedings of the National Academy of Sciences of the United States of America 101, no. 13 (2004): 4435–4440. 15070736 10.1073/pnas.0306786101 PMC384765

[8] J. R. Buchan and R. Parker , “Eukaryotic Stress Granules: The Ins and Outs of Translation,” Molecular Cell 36, no. 6 (2009): 932–941. 20064460 10.1016/j.molcel.2009.11.020 PMC2813218

[9] E. W. Wallace , J. L. Kear‐Scott , E. V. Pilipenko , et al., “Reversible, Specific, Active Aggregates of Endogenous Proteins Assemble Upon Heat Stress,” Cell 162, no. 6 (2015): 1286–1298. 26359986 10.1016/j.cell.2015.08.041 PMC4567705

[10] T. M. Franzmann and S. Alberti , “Protein Phase Separation as a Stress Survival Strategy,” Cold Spring Harbor Perspectives in Biology 11, no. 6 (2019): a034058. 30617047 10.1101/cshperspect.a034058 PMC6546044

[11] P. Gallardo , S. Salas‐Pino , and R. R. Daga , “Reversible Protein Aggregation as Cytoprotective Mechanism Against Heat Stress,” Current Genetics 67, no. 6 (2021): 849–855. 34091720 10.1007/s00294-021-01191-2 PMC8592950

[12] W. J. Welch and J. P. Suhan , “Morphological Study of the Mammalian Stress Response: Characterization of Changes in Cytoplasmic Organelles, Cytoskeleton, and Nucleoli, and Appearance of Intranuclear Actin Filaments in Rat Fibroblasts After Heat‐Shock Treatment,” Journal of Cell Biology 101, no. 4 (1985): 1198–1211. 3900086 10.1083/jcb.101.4.1198 PMC2113902

[13] R. M. Rivera , K. L. Kelley , G. W. Erdos , and P. J. Hansen , “Alterations in Ultrastructural Morphology of Two‐Cell Bovine Embryos Produced In Vitro and In Vivo Following a Physiologically Relevant Heat Shock,” Biology of Reproduction 69, no. 6 (2003): 2068–2077. 12930717 10.1095/biolreprod.103.020347

[14] Z. Török , I. Horváth , P. Goloubinoff , et al., “Evidence for a Lipochaperonin: Association of Active Proteinfolding GroESL Oligomers With Lipids Can Stabilize Membranes Under Heat Shock Conditions,” Proceedings of the National Academy of Sciences 94, no. 6 (1997): 2192–2197. 10.1073/pnas.94.6.2192 PMC20063 9122170

[15] L. Vigh , B. Maresca , and J. L. Harwood , “Does the Membrane's Physical State Control the Expression of Heat Shock and Other Genes?,” Trends in Biochemical Sciences 23, no. 10 (1998): 369–374. 9810221 10.1016/s0968-0004(98)01279-1

[16] G. Balogh , I. Horváth , E. Nagy , et al., “The Hyperfluidization of Mammalian Cell Membranes Acts as a Signal to Initiate the Heat Shock Protein Response,” FEBS Journal 272, no. 23 (2005): 6077–6086. 16302971 10.1111/j.1742-4658.2005.04999.x

[17] K. Richter , M. Haslbeck , and J. Buchner , “The Heat Shock Response: Life on the Verge of Death,” Molecular Cell 40, no. 2 (2010): 253–266. 20965420 10.1016/j.molcel.2010.10.006

[18] K. Ahmed , Y. Tabuchi , and T. Kondo , “Hyperthermia: An Effective Strategy to Induce Apoptosis in Cancer Cells,” Apoptosis 20, no. 11 (2015): 1411–1419. 26354715 10.1007/s10495-015-1168-3

[19] N. R. Datta , S. G. Ordóñez , U. S. Gaipl , et al., “Local Hyperthermia Combined With Radiotherapy and−/or Chemotherapy: Recent Advances and Promises for the Future,” Cancer Treatment Reviews 41, no. 9 (2015): 742–753. 26051911 10.1016/j.ctrv.2015.05.009

[20] M. A. Nashiry , G. R. A. Froemming , Y. S. Keong , A. B. M. Ismail , A. M. Din , and A. M. al‐Khateeb , “Severe Hyperthermia Induces Apoptosis Mediated by Caspases Activation and Suppression of Hsp90‐Alpha Expression in Osteosarcoma Cells,” Indonesian Biomedical Journal 11, no. 2 (2019): 167–174.

[21] E. M. Scutigliani , Y. Liang , H. Crezee , R. Kanaar , and P. M. Krawczyk , “Modulating the Heat Stress Response to Improve Hyperthermia‐Based Anticancer Treatments,” Cancers 13, no. 6 (2021): 1243. 33808973 10.3390/cancers13061243 PMC8001574

[22] F. Ritossa , “A New Puffing Pattern Induced by Temperature Shock and DNP in Drosophila,” Experientia 18, no. 12 (1962): 571–573.

[23] M. Ashburner and J. J. Bonner , “The Induction of Gene Activity in Drosophila by Heat Shock,” Cell 17, no. 2 (1979): 241–254. 110462 10.1016/0092-8674(79)90150-8

[24] N. S. Peterson , G. Moller , and H. K. Mitchell , “Genetic Mapping of the Coding Regions for Three Heat‐Shock Proteins in *Drosophila Melanogaster* ,” Genetics 92, no. 3 (1979): 891–902. 119669 10.1093/genetics/92.3.891 PMC1214044

[25] K. D. Sarge , V. Zimarino , K. Holm , C. Wu , and R. I. Morimoto , “Cloning and Characterization of Two Mouse Heat Shock Factors With Distinct Inducible and Constitutive DNA‐Binding Ability,” Genes & Development 5, no. 10 (1991): 1902–1911. 1717345 10.1101/gad.5.10.1902

[26] J. T. Westwood and C. Wu , “Activation of Drosophila Heat Shock Factor: Conformational Change Associated With a Monomer‐To‐Trimer Transition,” Molecular and Cellular Biology 13, no. 6 (1993): 3481–3486. 8497263 10.1128/mcb.13.6.3481-3486.1993 PMC359817

[27] K. D. Sarge , S. P. Murphy , and R. I. Morimoto , “Activation of Heat Shock Gene Transcription by Heat Shock Factor 1 Involves Oligomerization, Acquisition of DNA‐Binding Activity, and Nuclear Localization and Can Occur in the Absence of Stress,” Molecular and Cellular Biology 13, no. 3 (2023): 1392–1407. 10.1128/mcb.13.3.1392-1407.1993 PMC359449 8441385

[28] S. Lindquist , “The Heat‐Shock Response,” Annual Review of Biochemistry 55, no. 1 (1986): 1151–1191. 10.1146/annurev.bi.55.070186.005443 2427013

[29] E. T. Powers , R. I. Morimoto , A. Dillin , J. W. Kelly , and W. E. Balch , “Biological and Chemical Approaches to Diseases of Proteostasis Deficiency,” Annual Review of Biochemistry 78, no. 1 (2009): 959–991. 10.1146/annurev.biochem.052308.114844 19298183

[30] T. A. Allen , S. von Kaenel , J. A. Goodrich , and J. F. Kugel , “The SINE‐Encoded Mouse B2 RNA Represses mRNA Transcription in Response to Heat Shock,” Nature Structural & Molecular Biology 11, no. 9 (2004): 816–821. 10.1038/nsmb813 15300240

[31] P. D. Mariner , R. D. Walters , C. A. Espinoza , et al., “Human Alu RNA Is a Modular Transacting Repressor of mRNA Transcription During Heat Shock,” Molecular Cell 29, no. 4 (2008): 499–509. 18313387 10.1016/j.molcel.2007.12.013

[32] P. Yakovchuk , J. A. Goodrich , and J. F. Kugel , “B2 RNA and Alu RNA Repress Transcription by Disrupting Contacts Between RNA Polymerase II and Promoter DNA Within Assembled Complexes,” Proceedings of the National Academy of Sciences 106, no. 14 (2009): 5569–5574. 10.1073/pnas.0810738106 PMC2667051 19307572

[33] A. De Benedetti and C. Baglioni , “Activation of Hemin‐Regulated Initiation Factor‐2 Kinase in Heat‐Shocked HeLa Cells,” Journal of Biological Chemistry 261, no. 1 (1986): 338–342. 3941080

[34] R. L. Matts , Z. Xu , J. K. Pal , and J. J. Chen , “Interactions of the Heme‐Regulated eIF‐2 Alpha Kinase With Heat Shock Proteins in Rabbit Reticulocyte Lysates,” Journal of Biological Chemistry 267, no. 25 (1992): 18160–18167. 1355482

[35] R. L. Matts , R. Hurst , and Z. Xu , “Denatured Proteins Inhibit Translation in Hemin‐Supplemented Rabbit Reticulocyte Lysate by Inducing the Activation of the Heme‐Regulated eIF‐2.Alpha. Kinase,” Biochemistry 32, no. 29 (2002): 7323–7328. 10.1021/bi00080a001 8101730

[36] M. Holcik and N. Sonenberg , “Translational Control in Stress and Apoptosis,” Nature Reviews. Molecular Cell Biology 6, no. 4 (2005): 318–327. 15803138 10.1038/nrm1618

[37] P. Anderson and N. Kedersha , “Stressful Initiations,” Journal of Cell Science 115, no. 16 (2002): 3227–3234. 12140254 10.1242/jcs.115.16.3227

[38] N. Kedersha , G. Stoecklin , M. Ayodele , et al., “Stress Granules and Processing Bodies Are Dynamically Linked Sites of mRNP Remodeling,” Journal of Cell Biology 169, no. 6 (2005): 871–884. 15967811 10.1083/jcb.200502088 PMC2171635

[39] P. Anderson and N. Kedersha , “Stress Granules: The Tao of RNA Triage,” Trends in Biochemical Sciences 33, no. 3 (2008): 141–150. 18291657 10.1016/j.tibs.2007.12.003

[40] D. Mateju , T. M. Franzmann , A. Patel , et al., “An Aberrant Phase Transition of Stress Granules Triggered by Misfolded Protein and Prevented by Chaperone Function,” EMBO Journal 36, no. 12 (2017): 1669–1687. 28377462 10.15252/embj.201695957 PMC5470046

[41] H. A. Parag , B. Raboy , and R. G. Kulka , “Effect of Heat Shock on Protein Degradation in Mammalian Cells: Involvement of the Ubiquitin System,” EMBO Journal 6, no. 1 (1987): 55–61. 3034579 10.1002/j.1460-2075.1987.tb04718.x PMC553356

[42] J. R. Buchan , R. M. Kolaitis , J. P. Taylor , and R. Parker , “Eukaryotic Stress Granules Are Cleared by Autophagy and Cdc48/VCP Function,” Cell 153, no. 7 (2013): 1461–1474. 23791177 10.1016/j.cell.2013.05.037 PMC3760148

[43] K. Dokladny , M. N. Zuhl , M. Mandell , et al., “Regulatory Coordination Between Two Major Intracellular Homeostatic Systems: Heat Shock Response and Autophagy,” Journal of Biological Chemistry 288, no. 21 (2013): 14959–14972. 23576438 10.1074/jbc.M113.462408 PMC3663517

[44] K. Dokladny , O. B. Myers , and P. L. Moseley , “Heat Shock Response and Autophagy—Cooperation and Control,” Autophagy 11, no. 2 (2015): 200–213. 25714619 10.1080/15548627.2015.1009776 PMC4502786

[45] R. W. Walters , D. Muhlrad , J. Garcia , and R. Parker , “Differential Effects of Ydj1 and Sis1 on Hsp70‐Mediated Clearance of Stress Granules in *Saccharomyces cerevisiae* ,” RNA 21, no. 9 (2015): 1660–1671. 26199455 10.1261/rna.053116.115 PMC4536325

[46] N. Jain , A. Rai , R. Mishra , and S. Ganesh , “Loss of Malin, but Not Laforin, Results in Compromised Autophagic Flux and Proteasomal Dysfunction in Cells Exposed to Heat Shock,” Cell Stress & Chaperones 22, no. 2 (2017): 307–315. 27975203 10.1007/s12192-016-0754-9 PMC5352594

[47] C. Vigouroux , M. Auclair , E. Dubosclard , et al., “Nuclear Envelope Disorganization in Fibroblasts From Lipodystrophic Patients With Heterozygous R482Q/W Mutations in the Lamin A/C Gene,” Journal of Cell Science 114, no. Pt 24 (2001): 4459. 11792811 10.1242/jcs.114.24.4459

[48] A. Muchir , B. G. van Engelen , M. Lammens , et al., “Nuclear Envelope Alterations in Fibroblasts From LGMD1B Patients Carrying Nonsense Y259X Heterozygous or Homozygous Mutation in Lamin A/C Gene,” Experimental Cell Research 291, no. 2 (2003): 352–362. 14644157 10.1016/j.yexcr.2003.07.002

[49] H. J. Worman , L. G. Fong , A. Muchir , and S. G. Young , “Laminopathies and the Long Strange Trip From Basic Cell Biology to Therapy,” Journal of Clinical Investigation 119, no. 7 (2009): 1825–1836. 19587457 10.1172/JCI37679 PMC2701866

[50] M. Stiekema , M. A. M. J. van Zandvoort , F. C. S. Ramaekers , and J. L. V. Broers , “Structural and Mechanical Aberrations of the Nuclear Lamina in Disease,” Cells 9, no. 8 (2020): 1884. 32796718 10.3390/cells9081884 PMC7464082

[51] A. K. Velichko , E. N. Markova , N. V. Petrova , S. V. Razin , and O. L. Kantidze , “Mechanisms of Heat Shock Response in Mammals,” Cellular and Molecular Life Sciences 70, no. 22 (2013): 4229–4241. 23633190 10.1007/s00018-013-1348-7 PMC11113869

[52] D. A. Fletcher and R. D. Mullins , “Cell Mechanics and the Cytoskeleton,” Nature 463, no. 7280 (2010): 485–492. 20110992 10.1038/nature08908 PMC2851742

[53] M. Li , L. Peng , Z. Wang , et al., “Roles of the Cytoskeleton in Human Diseases,” Molecular Biology Reports 50, no. 3 (2023): 2847–2856. 36609753 10.1007/s11033-022-08025-5

[54] M. Crisp , Q. Liu , K. Roux , et al., “Coupling of the Nucleus and Cytoplasm: Role of the LINC Complex,” Journal of Cell Biology 172, no. 1 (2006): 41–53. 16380439 10.1083/jcb.200509124 PMC2063530

[55] M. L. Lombardi , D. E. Jaalouk , C. M. Shanahan , B. Burke , K. J. Roux , and J. Lammerding , “The Interaction Between Nesprins and Sun Proteins at the Nuclear Envelope Is Critical for Force Transmission Between the Nucleus and Cytoskeleton,” Journal of Biological Chemistry 286, no. 30 (2011): 26743–26753. 21652697 10.1074/jbc.M111.233700 PMC3143636

[56] M. B. Omary , N. O. Ku , G. Z. Tao , D. M. Toivola , and J. Liao , ““Heads and Tails” of Intermediate Filament Phosphorylation: Multiple Sites and Functional Insights,” Trends in Biochemical Sciences 31, no. 7 (2006): 383–394. 16782342 10.1016/j.tibs.2006.05.008

[57] B. MacTaggart and A. Kashina , “Posttranslational Modifications of the Cytoskeleton,” Cytoskeleton 78, no. 4 (2021): 142–173. 34152688 10.1002/cm.21679 PMC8856578

[58] M. L. Styers , G. Salazar , R. Love , A. A. Peden , A. P. Kowalczyk , and V. Faundez , “The Endo‐Lysosomal Sorting Machinery Interacts With the Intermediate Filament Cytoskeleton,” Molecular Biology of the Cell 15, no. 12 (2004): 5369–5382. 15456899 10.1091/mbc.E04-03-0272 PMC532017

[59] D. M. Toivola , G. Z. Tao , A. Habtezion , J. Liao , and M. B. Omary , “Cellular Integrity Plus: Organelle‐Related and Protein‐Targeting Functions of Intermediate Filaments,” Trends in Cell Biology 15, no. 11 (2005): 608–617. 16202602 10.1016/j.tcb.2005.09.004

[60] N. Schwarz and R. E. Leube , “Intermediate Filaments as Organizers of Cellular Space: How They Affect Mitochondrial Structure and Function,” Cells 5, no. 3 (2016): 30. 27399781 10.3390/cells5030030 PMC5040972

[61] S. Etienne‐Manneville , “Cytoplasmic Intermediate Filaments in Cell Biology,” Annual Review of Cell and Developmental Biology 34 (2018): 1–28. 10.1146/annurev-cellbio-100617-062534 30059630

[62] W. H. Goldmann , “Intermediate Filaments and Cellular Mechanics,” Cell Biology International 42, no. 2 (2018): 132–138. 28980752 10.1002/cbin.10879

[63] K. A. Spriggs , M. Bushell , and A. E. Willis , “Translational Regulation of Gene Expression During Conditions of Cell Stress,” Molecular Cell 40, no. 2 (2010): 228–237. 20965418 10.1016/j.molcel.2010.09.028

[64] O. L. Kantidze , A. K. Velichko , and S. V. Razin , “Heat Stress‐Induced Transcriptional Repression,” Biochemistry 80, no. 8 (2015): 990–993. 26547066 10.1134/S0006297915080039

[65] M. E. Tanenbaum , L. A. Gilbert , L. S. Qi , J. S. Weissman , and R. D. Vale , “A Protein‐Tagging System for Signal Amplification in Gene Expression and Fluorescence Imaging,” Cell 159, no. 3 (2014): 635–646. 25307933 10.1016/j.cell.2014.09.039 PMC4252608

[66] D. C. Dieterich , A. J. Link , J. Graumann , D. A. Tirrell , and E. M. Schuman , “Selective Identification of Newly Synthesized Proteins in Mammalian Cells Using Bioorthogonal Noncanonical Amino Acid Tagging (BONCAT),” Proceedings of the National Academy of Sciences 103, no. 25 (2006): 9482–9487. 10.1073/pnas.0601637103 PMC1480433 16769897

[67] R. M. Vabulas , S. Raychaudhuri , M. Hayer‐Hartl , and F. U. Hartl , “Protein Folding in the Cytoplasm and the Heat Shock Response,” Cold Spring Harbor Perspectives in Biology 2, no. 12 (2010): a004390. 21123396 10.1101/cshperspect.a004390 PMC2982175

[68] T. Gidalevitz , V. Prahlad , and R. I. Morimoto , “The Stress of Protein Misfolding: From Single Cells to Multicellular Organisms,” Cold Spring Harbor Perspectives in Biology 3, no. 6 (2011): a009704. 21536706 10.1101/cshperspect.a009704 PMC3098679

[69] S. S. Lam , J. D. Martell , K. J. Kamer , et al., “Directed Evolution of APEX2 for Electron Microscopy and Proximity Labeling,” Nature Methods 12, no. 1 (2014): 51–54. 25419960 10.1038/nmeth.3179 PMC4296904

[70] B. J. DiDomenico , G. E. Bugaisky , and S. Lindquist , “The Heat Shock Response Is Self‐Regulated at Both the Transcriptional and Posttranscriptional Levels,” Cell 31 (1982): 593–603. 7159929 10.1016/0092-8674(82)90315-4

[71] P. Workman , F. Burrows , L. E. Neckers , et al., “Drugging the Cancer Chaperone HSP90: Combinatorial Therapeutic Exploitation of Oncogene Addiction and Tumor Stress,” Annals of the New York Academy of Sciences 1113 (2007): 202–216. 17513464 10.1196/annals.1391.012

[72] S. K. Calderwood and A. Murshid , “Molecular Chaperone Accumulation in Cancer and Decrease in Alzheimer's Disease: The Potential Roles of HSF1,” Frontiers in Neuroscience 11 (2017): 192. 28484363 10.3389/fnins.2017.00192 PMC5399083

[73] P. Thomas and T. G. Smart , “HEK293 Cell Line: A Vehicle for the Expression of Recombinant Proteins,” Journal of Pharmacological and Toxicological Methods 51, no. 3 (2005): 187–200. 15862464 10.1016/j.vascn.2004.08.014

[74] M. Uhlen , L. Fagerberg , B. M. Hallström , et al., “Proteomics. Tissue‐Based Map of the Human Proteome,” Science 347, no. 6220 (2015): 1260419. 25613900 10.1126/science.1260419

[75] M. Uhlen , C. Zhang , S. Lee , et al., “A Pathology Atlas of the Human Cancer Transcriptome,” Science 357, no. 6352 (2017): eaan2507. 28818916 10.1126/science.aan2507

[76] J. Lammerding , L. G. Fong , J. Y. Ji , et al., “Lamins A and C but Not Lamin B1 Regulate Nuclear Mechanics,” Journal of Biological Chemistry 281, no. 35 (2006): 25768–25780. 16825190 10.1074/jbc.M513511200

[77] M. Guo , I. Y. Wong , A. S. Moore , et al., “Vimentin Intermediate Filaments as Structural and Mechanical Coordinators of Mesenchymal Cells,” Nature Cell Biology 27, no. 8 (2025): 1210–1218. 40764390 10.1038/s41556-025-01713-x

[78] Y. Tang , J. Ren , and C. C. Li , “Establishment of a GFP::LMNB1 Knockin Cell Line (CSUi002‐A‐1) From a Dystonia Patient‐Specific iPSC by CRISPR/Cas9 Editing,” Stem Cell Research 55 (2021): 102505. 34438319 10.1016/j.scr.2021.102505

