# Supplementary material for: Heat Stress Triggers Nuclear Invagination and Spatial Compartmentalization of Protein Metabolism
Source: Cell Prolif. 2026 Mar 12;59(8):e70196. doi: 10.1111/cpr.70196 (PMC13325906; doi:10.1111/cpr.70196)
Supplement: Supplementary file 1 [file CPR-59-e70196-s003.docx]

**Supplementary Figures**

**Supplementary Figure 1. Heat stress induced nuclear invagination demonstrate graduality, generality and reversibility.**

A. Immunofluorescence of HEK293T cells (stained with Lamin B1 and DAPI) showing nuclear morphology alterations under different heat stress temperature gradients (37°C, 40°C, 43°C; 2 h treatment). Scale bars: 25 μm.

B. Immunofluorescence of HEK293T cells (stained with Lamin B1 and DAPI) showing nuclear morphology alterations under different heat stress duration gradients (43°C; 10 min, 30 min, 1 h, 3 h treatment). Scale bars: 20 μm.

C. Immunofluorescence of HeLa cells (stained with Lamin B1 and DAPI) showing nuclear morphology alterations under heat stress (43°C, 3 h). Scale bars: 10 μm.

D. Immunofluorescence of Huh7 cells (stained with Lamin B1 and DAPI) showing nuclear morphology alterations under heat stress (43°C, 3 h). Scale bars: 10 μm.

E. Flow cytometry analysis of apoptotic cell proportion via Propidium Iodide (PI) staining upon heat stress (43°C; 1 h, 3 h).

F. Immunofluorescence of HEK293T cells (stained with Lamin B1 and DAPI) showing nuclear morphology alterations during recovery (Timepoints: 0, 3 , 6 , 20 h) after 1h heat stress at 43°C. Scale bars: 50 μm.

G. Schematic illustration of LMNB1 fluorescent reporter cell line generation.

H. Live-cell imaging of nuclear invaginations in LMNB1 fluorescent reporter cell line upon heat stress (43°C; 1 h). Scale bars: 2.5 μm.

**Supplementary Figure 2. Cytoskeletal remodeling and organelle reorganization during nuclear stress adaptation.**

A.	Immunofluorescence of HeLa cells stained with anti-β-actin (actin, red), Lamin B1 (nuclear membrane, green) and DAPI (nuclei, blue), showing actin distribution following heat stress (HS, 43°C for 3 h) compared to untreated cells (NC). Scale bars: 10 μm.

B. Quantification of β-actin fluorescence intensity, showing the distribution across total cellular, nuclear, and invagination regions. (n = 12)

C. Immunofluorescence of HeLa cells stained with anti-β-tubulin (tubulin, red), Lamin B1 (nuclear membrane, green) and DAPI (nuclei, blue), showing tubulin distribution following heat stress (HS, 43°C for 3 h) compared to untreated cells (NC). Scale bars: 10 μm.

D. Quantification of β-tubulin fluorescence intensity, showing the distribution across total cellular, nuclear, and invagination regions. (n = 17)

E.	Immunofluorescence of HEK293T cells stained with anti-SUN1 (LINC complex, green), Lamin B1 (nuclear membrane, red) and DAPI (nuclei, blue), showing SUN1 distribution following heat stress (HS, 43°C for 1 h) compared to untreated cells (NC). Scale bars: 5 μm.

F.	Immunofluorescence of HEK293T cells stained with anti-NUP153 (nuclear pore complex, green), Lamin B1 (nuclear membrane, red) and DAPI (nuclei, blue), showing NUP153 distribution following heat stress (HS, 43°C for 1 h) compared to untreated cells (NC). Scale bars: 5 μm.

G. Immunofluorescence of HEK293T cells stained with anti-TOM20 (mitochondria, red), Lamin B1 (nuclear membrane, green) and DAPI (nuclei, blue), showing mitochondrial enrichment in nuclear invagination regions upon heat stress (HS, 43°C for 1 h) and their distribution during recovery (H1R6: 6 h recovery, H1R18: 18 h recovery). Scale bars: 10 μm.

H. Immunofluorescence of HeLa cells stained with anti-eIF6 (red), Lamin B1 (nuclear membrane, green) and DAPI (nuclei, blue), showing nucleoli disassembly and diffusion following heat stress (HS, 43°C for 3 h) compared to untreated cells (NC). Scale bars: 10 μm.

Statistical analysis: Data in B and D were analyzed using one-way ANOVA followed by Tukey's post-hoc test for multiple comparisons. The significance labels are defined in the Figure 1 legend.

**Supplementary Figure 3. The impact of candidate factors perturbation on nuclear invagination phenotype under heat stress.**

A.	Knockdown efficiency of candidate genes detected by RT-qPCR. Relative mRNA levels were calculated using the 2^^-ΔΔCt^ method and are presented relative to wild type cells (WT). Certain housekeeping genes exhibiting limited knockdown efficiency.

B.	Expression levels of candidate genes after overexpression detected by RT-qPCR. Relative mRNA levels were calculated using the 2^^-ΔΔCt^ method and are presented relative to WT.

C.	Changes in nuclear circularity before and after heat stress (43°C-1h) following perturbation of candidate factors. Heat stress induces a significant reduction in nuclear circularity across all perturbations. (The number of cells analyzed per condition is indicated on the graphs).

D.	Changes in the proportion of nuclei displaying invagination before and after heat stress (43°C-1h) following candidate factor perturbation. Heat stress induces a significant increase in the proportion of nuclei showing invagination across all perturbations.

Statistical analysis: Data in C and D were analyzed using one-way ANOVA followed by Tukey's post-hoc test for multiple comparisons. The significance labels are defined in the Figure 1 legend.

**Supplementary Figure 4. Quality control and systems analysis of newly synthesized proteome profiling.**

A. Immunofluorescence of HEK293T cells stained with anti-RPS3 (ribosome, red), Lamin B1 (nuclear membrane, green) and DAPI (nuclei, blue), showing ribosome distribution following heat stress (HS, 43°C for 1 h) compared to untreated cells (NC). Scale bars: 5 μm.

B. Immunofluorescence of HEK293T cells stained with anti-RPL18A (ribosome, green), Lamin B1 (nuclear membrane, red) and DAPI (nuclei, blue), showing ribosome distribution following heat stress (HS, 43°C for 1 h) compared to untreated cells (NC). Scale bars: 5 μm.

C. Immunofluorescence of HEK293T cells stained with anti-eIF2α (translation initiation complex, green), Lamin B1 (nuclear membrane, red) and DAPI (nuclei, blue), showing translation initiation complex distribution following heat stress (HS, 43°C for 1 h) compared to untreated cells (NC). Scale bars: 5 μm.

D. Representative SunTag images of HEK293T cells expressing the Kif18b-24×GCN4 reporter and scFv-sfGFP. Following heat stress, no evident clustering of active translation sites is observed; instead, distinct sfGFP aggregates form, which likely represent misfolded protein accumulations in invagations. Scale bars: 5 μm.

E. Bar plot showing the number of proteins identified in each newly synthesized proteome sample across different conditions (HS: Heat Stress, ctrl: Negative Control, NC: Untreated) and three biological replicates.

F. Principal Component Analysis (PCA) plot of newly synthesized proteome profiles.

G. Hierarchical clustering and correlation heat map showing the Pearson correlation coefficients among all newly synthesized proteome samples.

H. Heat map illustrating the expression levels of the newly synthesized proteome across all experimental conditions (HS, ctrl, NC) and biological replicates.

I. Western blot analysis demonstrating the efficiency of HPG-mediated biotin conjugation at varying HPG incorporation concentrations (0-2 mM).

J. Western blot analysis evaluating the efficiency of the streptavidin pulldown process. Lanes represent Input (IN), Flow Through (FL), and Immunoprecipitation/Pulldown (IP).

K. Immunofluorescence of HEK293T cells stained with anti-ATP5L (green), Lamin B1 (nuclear membrane, red) and DAPI (nuclei, blue), showing the accumulation of ATP5L following heat stress (HS, 43°C for 1 h) compared to untreated cells (NC). Scale bars: 5 μm.

L. Immunofluorescence of HEK293T cells stained with anti-PRDX4 (green), Lamin B1 (nuclear membrane, red) and DAPI (nuclei, blue), showing the accumulation of PRDX4 following heat stress (HS, 43°C for 1 h) compared to untreated cells (NC). Scale bars: 5 μm.

M. Immunofluorescence of HEK293T cells stained with anti-PSMB6 (green), Lamin B1 (nuclear membrane, red) and DAPI (nuclei, blue), showing PSMB6 distribution following heat stress (HS, 43°C for 1 h) compared to untreated cells (NC). Scale bars: 5 μm.

**Supplementary Figure 5. Quality control and systems analysis of proximity labeling proteome profiling.**

A. Immunofluorescence of HeLa cells stained with anti-LAMP1 (lysosome, red), Lamin B1 (nuclear membrane, green) and DAPI (nuclei, blue), showing lysosomal redistribution following heat stress (HS, 43°C for 3 h) compared to untreated cells (NC). Scale bars: 10 μm.

B. Immunofluorescence showing biotinylation in nuclear invagination regions by proximity labeling. The red signal (anti-FLAG staining) indicates the localization of the APEX2 proximity labeling enzyme, and the yellow signal (Streptavidin-Alexa 594) marks the biotinylated proximal proteins. Scale bars: 5 μm.

C. Bar plot showing the number of proteins identified in each proximity labeling proteome sample across different conditions (NC: NES-NC, NH: NES-HS, VC: VIM-NC, VH: VIM-HS) and three biological replicates.

D. Principal Component Analysis (PCA) plot of proximity labeling proteome profiles.(NC: NES-NC, NH: NES-HS, VC: VIM-NC, VH: VIM-HS)

E. Hierarchical clustering and correlation heat map showing the Pearson correlation coefficients among all proximity labeling proteome samples (NC: NES-NC, NH: NES-HS, VC: VIM-NC, VH: VIM-HS).

F. Western blot analysis evaluating the efficiency of the streptavidin pulldown process. Lanes represent Input (IN), Flow Through (FL), and Immunoprecipitation/Pulldown (IP)

G. Heat map illustrating the expression levels of the proximity labeling proteome across all experimental conditions (NC: NES-NC, NH: NES-HS, VC: VIM-NC, VH: VIM-HS) and biological replicates.

H. Immunofluorescence of HEK293T cells stained with anti-Ubiquitin (Ubiquitin, red), Lamin B1 (nuclear membrane, green) and DAPI (nuclei, blue), showing ubiquitin distribution following heat stress (HS, 43°C for 1 h) compared to untreated cells (NC). Scale bars: 5 μm.

I. Immunofluorescence of HEK293T cells stained with anti-TOM20 (mitochondrial, red), Lamin B1 (nuclear membrane, green) and DAPI (nuclei, blue), showing mitochondrial enrichment in nuclear invagination regions under MG132 treatment (10 μM, 20 h). Scale bars: 25 μm.

J. Immunofluorescence of HEK293T cells (stained with Lamin B1 and DAPI) illustrating the modulation of nuclear invagination formation. Representative images display the nuclear invagination under MG132 (10 μM for 20 h), heat stress (HS, 43°C for 1 h) or BTZ (1 μM for 20 h) induction, and the reversal of the phenotype by Cycloheximide (CHX, 100 μg/ml for 20 h) combined with either MG132, heat stress or BTZ. Scale bars: 5 μm.

K. Immunofluorescence of Hela cells (stained with Lamin B1 and DAPI) illustrating the modulation of nuclear invagination formation. Representative images display the nuclear invagination under MG132 (10 μM for 20 h) or heat stress (HS, 43°C for 1 h) induction and the reversal of the phenotype by Cycloheximide (CHX, 100 μg/ml for 20 h) combined with either MG132 or heat stress. Scale bars: 5 μm.

L. Immunofluorescence of Huh7 cells (stained with Lamin B1 and DAPI) illustrating the modulation of nuclear invagination formation. Representative images display the nuclear invagination under MG132 (10 μM for 20 h) or heat stress (HS, 43°C for 1 h) induction and the reversal of the phenotype by Cycloheximide (CHX, 100 μg/ml for 20 h) combined with either MG132 or heat stress. Scale bars: 5 μm.

**Supplementary Figure S6. Kinetics of the canonical heat shock response during heat stress and recovery.**

A. Dot plot showing the enrichment significance (-log_10_ (P value)) of selected Gene Ontology (GO) biological processes related to the canonical heat shock response, at different time points relative to NC. The size of each dot corresponds to the count of genes mapped to the respective GO term. Time points: heat stress (H1: 43°C for 1 h) and subsequent recovery phases at 37°C (H1R1: 1 h recovery; H1R6: 6 h recovery; H1R18: 18 h recovery). The enrichment significance and count for these pathways increase substantially during the later recovery phases (H1R6 and H1R18).

B. Heatmap showing the expression changes relative to NC of a curated protein set derived from heat shock-related GO terms. Each row represents a protein, and each column represents a condition (H1, H1R1, H1R6, H1R18).

C. Bar graph showing the expression changes relative to NC of three representative canonical HSPs (HSPA1A, HSP90AA1, DNAJB1) under the indicated conditions. Their pronounced upregulation is evident primarily during the recovery phase.
